# Targeting of the CD161 Inhibitory Receptor Enhances Bone‐Marrow‐Resident Memory CD8^+^ T‐Cell‐Mediated Immunity against Multiple Myeloma

**DOI:** 10.1002/advs.202510888

**Published:** 2025-08-25

**Authors:** Liwen Wang, Linzhi Xie, Yi Zhou, Shiming Tan, Yan Yu, Qin Zhang, Xuefeng Chen, Yuhan Yan, Ruiheng Luo, Han Xiao, Qian Cheng, Jian Zhang, Ying Li, Erhua Wang, Tiebin Jiang, Jing Liu, Xin Li

**Affiliations:** ^1^ Department of Hematology Third Xiangya Hospital Central South University Changsha Hunan Province 410013 China

**Keywords:** bone marrow microenvironment, CD161, multiple myeloma, tissue resident memory T cells

## Abstract

Immune checkpoint inhibitors (ICIs) have transformed the treatment of many solid tumors. Still, their effectiveness in multiple myeloma (MM) remains underwhelming, highlighting the need to explore alternative approaches beyond conventional ICIs. Here, CD161 is identified as a novel inhibitory receptor on bone marrow (BM) tissue resident memory CD8^+^ T cells (CD8^+^ TRM), known for their sustained presence and vital role in local immune surveillance in MM BM tumor microenvironments. The CD161–CLEC2D axis, where CD161 interacts with CLEC2D on MM cells, mediates immune suppression and TRM dysfunction. Blocking CD161 enhances TRM function, including tissue residency, proliferation, and antitumor activity. CD161 blockade significantly alleviates chimeric antigen receptor T‐cell (CAR‐T) exhaustion and enhances their antimyeloma function ex vivo. These findings identified the CD161–CLEC2D pathway as a potential novel target for immunotherapy of MM.

## Graphical abstract

CD161 is identified as a novel inhibitory receptor on bone marrow (BM)‐resident CD8⁺ T cells in multiple myeloma (MM). Through interaction with its ligand CLEC2D, CD161 impairs T‐cell function and promotes immune suppression. Blockade of CD161 restores tissue residency and cytotoxicity of CD8⁺ T cells and enhances chimeric antigen receptor T‐cell (CAR‐T) activity, providing a potential immunotherapeutic target in MM.

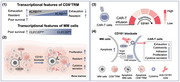

## 1 Introduction

Multiple myeloma (MM) is the second most prevalent hematologic malignancy and remains incurable. Despite advances in therapies, most patients eventually succumb to disease progression and complications.^[^ ^1^ ^]^ In patients with relapsed and refractory MM (RRMM), factors such as tumor heterogeneity and clonal selection, and the tumor microenvironment (TME), significantly hinder the effectiveness of MM treatments.^[^ ^2^ ^]^ Immune cell exhaustion and immunosuppressive TME are critical drivers for MM relapse and progression.^[^ ^2^, ^3^ ^]^ Unfortunately, the classical immune checkpoint inhibitors (ICIs) targeting PD‐1, LAG3, and CTLA‐4 have shown limited clinical efficacy in both newly diagnosed MM (NDMM) and RRMM.^[^ ^4^ ^]^

MM cells primarily infiltrate the bone marrow (BM), interacting with stromal and immune cells to establish an immunosuppressive TME.^[^ ^2^, ^5^ ^]^ Among BM immune cells, tissue resident memory CD8^+^ T cells (CD8^+^ TRM) play a crucial role in maintaining local immune surveillance and restraining tumor growth. The depletion of BM CD8^+^ TRM and increased expression of inhibitory receptors, such as PD‐1 and T cell immune receptor with Ig and ITIM domains (TIGIT), correlate with weakened immune surveillance in MM patients.^[^ ^6^ ^]^ However, single‐agent blockade targeting these pathways fails to effectively rescue TRM function.^[^ ^6b^ ^]^

Identifying the inhibitory receptors that govern BM CD8^+^ TRM exhaustion is of substantial clinical importance. Here, we conducted a meta‐analysis of BM CD8^+^ TRM across different disease stages integrating single‐cell RNA sequencing (scRNA‐seq) datasets from 17 healthy individuals and 83 patients. Our findings reveal that BM CD8^+^ TRM highly express the inhibitory receptor CD161, which interacts with its ligand CLEC2D on MM cells to suppress CD8^+^ TRM function. Blocking the CD161–CLEC2D interaction enhances the function of CD8^+^ TRM, including tissue residency, proliferation, cytotoxicity, and cytokine production. Importantly, CD161 blockade significantly alleviates chimeric antigen receptor T‐cell (CAR‐T) exhaustion and enhances their antimyeloma function ex vivo. Additionally, CD8^+^ TRM expressing CD161 are present in various hematologic malignancies and exhibit similar functional impairments. Our findings identified the CD161–CLEC2D pathway as a potential novel target for immunotherapy of MM.

## 2 Results

### 2.1 Transcriptional Features of BM CD8^+^ TRM Correlate with MM Disease Progression

**Figure 1:**
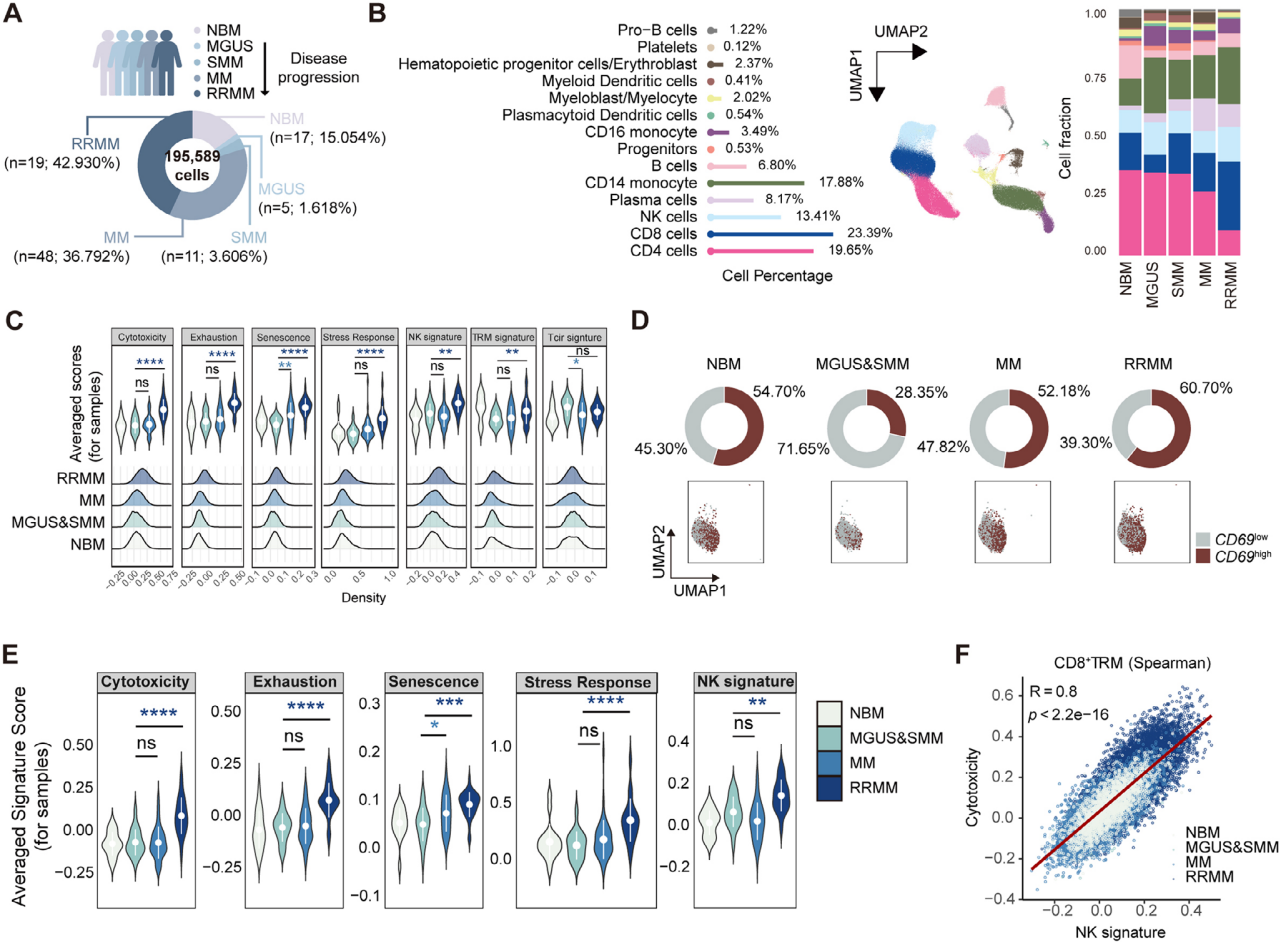
Transcriptional features of BM CD8^+^ TRM correlate with MM disease progression. A) A schematic depicting population classification, proportions included in the study, and the number of cells analyzed. B) UMAP was used to embed ≈195 589 cells from 100 individuals into a 2D space, color‐coded by cell type (center). The lollipop chart (left) displays the proportion of each cell type. Bar graphs (right) compare cell‐type distributions between the NBM and MM stages. C) Violin plots (top) and ridge plots (bottom) demonstrate the expression of seven curated gene signatures in CD8^+^ T cells across NBM and three MM stages (MGUS and SMM, MM, and RRMM). Comparisons between each MM stage and NBM are indicated. Data were presented as mean ± SD. Significance was calculated with one way analysis of variance (ANOVA). *p < 0.05, **p < 0.01, ****p < 0.0001, and ns, no significance. D) UMAP plots (bottom) and ring pie charts (top) depict the distribution and proportion of high and low *CD69‐*expression populations within CD8^+^ T cells across NBM and MM stages. E) Violin plot showing the expression of five curated gene signatures in CD8^+^ TRM across NBM and MM stages. Comparisons of each MM stage and NBM are indicated. Data were presented as mean ± SD. Significance was calculated with one way ANOVA. *p < 0.05, **p < 0.01, ***p < 0.001, ****p < 0.0001, and ns, no significance. F) Spearman correlation between NK signature scores (*x*‐axis) and cytotoxicity signature scores (*y*‐axis) in CD8^+^ TRM. Significance was calculated with Spearman test. UMAP, uniform manifold approximation and projection; NBM, normal bone marrow; MGUS, monoclonal gammopathy of undetermined significance; RRMM, relapsed and refractory multiple myeloma; MM, multiple myeloma; TRM, tissue resident memory.

To systematically analyze the BM–TME during the progression of MM, we first integrated publicly available BM scRNA‐seq data across normal bone marrow (NBM, *n* = 17) and four progression stages of MM, including monoclonal gammopathy of undetermined significance (MGUS, *n* = 5), smoldering multiple myeloma (SMM, *n* = 11), MM (*n* = 48), and RRMM (*n* = 19). In total, 195 589 high‐quality cells were identified following rigorous quality control (**Figure** **1**A), and 13 distinct cell types were classified for downstream analysis (Figure 1B; Figure S1A and Table S1, Supporting Information). CD4^+^ and CD8^+^ T cells constituted the most abundant subset, with an increase in CD8^+^ T‐cell fraction during the disease progression (from 7% in MGUS to 28% in RRMM) (Figure 1B). We next analyzed the functional changes of CD8^+^ T cells. Due to the limited cell numbers in MGUS and SMM, these two stages were merged into a single group. Compared to NBM, CD8^+^ T cells in MM and RRMM exhibited enhanced cytotoxicity, alongside increased exhaustion, senescence, stress responses, and natural killer (NK) cell signature (Figure 1C). Notably, CD8^+^ T cells in RRMM exhibited the highest TRM signature scores (Figure 1C), indicating that a greater proportion of CD8^+^ T cells in this stage possess tissue‐resident features. However, whether these cells remain functionally competent or are impaired remains unclear. Therefore, we subsequently performed an in‐depth phenotypic and functional analysis of CD8^+^ TRM cells within the BM.

First, we analyzed the correlation between TRM signature scores and several core TRM‐related genes (*CD69*, *CXCR3*, *CXCR6*, *ITGAE*, *GZMB*, *PRDM1*, *RUNX3*, and *SMAD3*) in CD8^+^ T cells. Among these, *CD69* showed the strongest association with higher TRM signature scores (Figure S1B, Supporting Information), consistent with its previously reported role as a marker for BM TRM.^[^ ^6^, ^7^ ^]^ CD8^+^ T cells were stratified into *CD69* ^high^ and *CD69* ^low^ subpopulations (Figure 1D). Consistently, *CD69* ^high^ cells exhibited higher TRM signature scores and lower circulating T‐cell (Tcir) signature scores (Figure S1C, Supporting Information). Subsequently, we validated the expression of CD69 on BM T cells at the protein level. Mononuclear cells from BM and peripheral blood (PB) of healthy controls (HC) and MM patients were analyzed by flow cytometry. Initial analysis identified a distinct cluster of BM T cells (Figure S1D, Supporting Information), which was almost absent in PB, suggesting that these cells are BM‐resident and do not circulate in PB. This cluster expressed CD3, CD8, and CD69, but neither CD4 nor CD103 (Figure S1E, Supporting Information). In all tested patient samples, CD8^+^CD69^+^CD103^–^ cells were almost exclusively restricted to BM (≈40%) and essentially absent from PB (Figure S1F, Supporting Information). Collectively, we identified that CD69^+^CD8^+^ T subsets were BM CD8^+^ TRM by integrating scRNA‐seq and flow cytometry data.

We next investigated the transcriptional features of the CD8^+^ TRM during the progression of MM. Compared to NBM, CD8^+^ TRM in MM and RRMM exhibited enhanced cytotoxicity, exhaustion, senescence, and stress‐response states (Figure 1E). Interestingly, higher cytotoxicity signature scores were correlated with elevated NK signature scores in CD8^+^ TRM (Figure 1F). This finding aligns with previous studies suggesting that the transition of CD8^+^ T cells to NK‐like T cells is associated with T‐cell dysfunction,^[^ ^8^ ^]^ highlighting a potential causal link between increased NK‐like characteristics and impaired TRM functionality in MM.

### 2.2 CD161/CLEC2D Axis Marks Dysfunctional CD8⁺ TRM State and Poor Prognosis in MM

**Figure 2:**
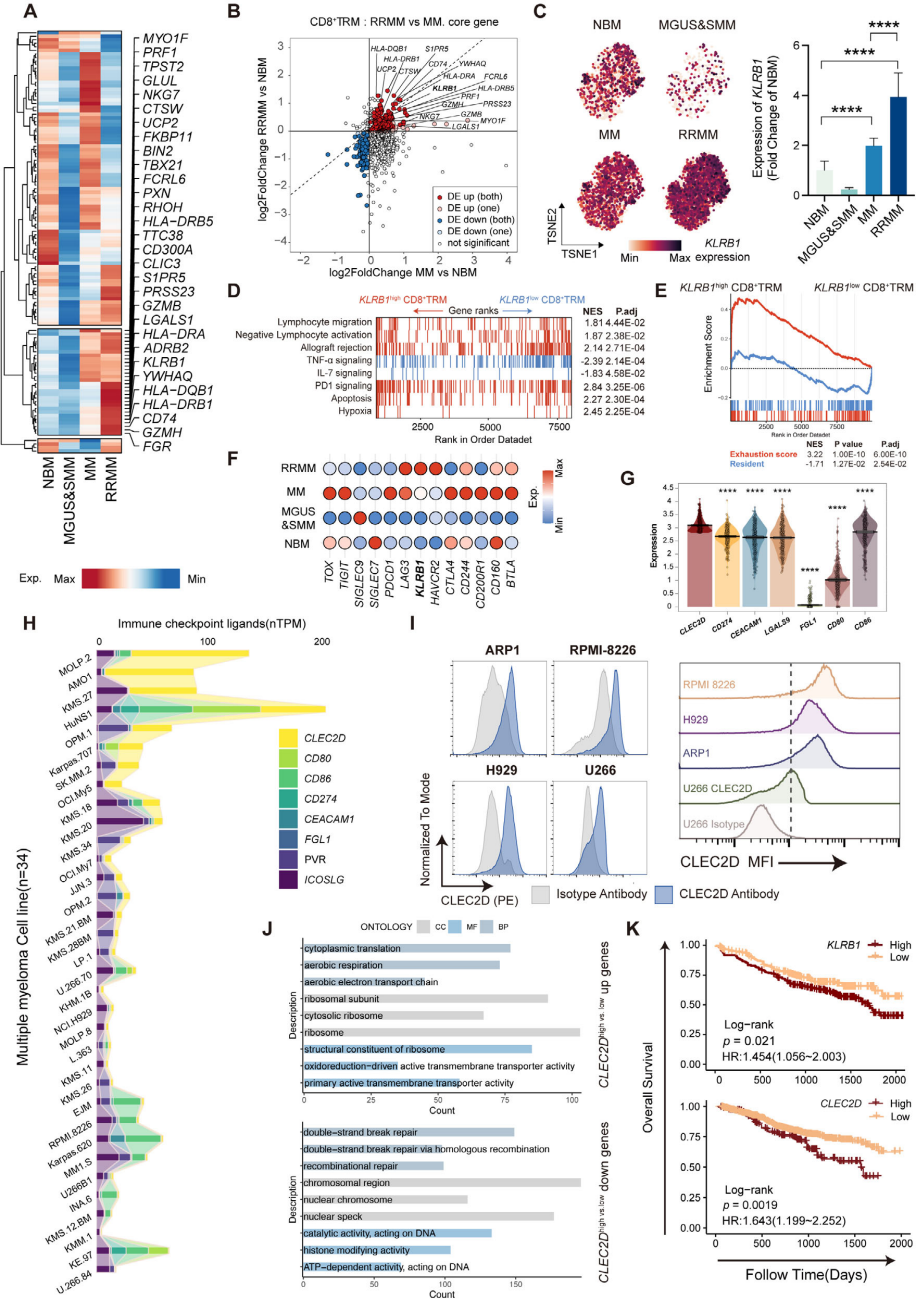
*KLRB1* mediates immunosuppressive and dysfunctional states in CD8^+^ TRM. A) Heatmap illustrating the relative expression of NK signature genes in CD8^+^ TRM across NBM and three MM stages. B) Double fold change plot depicting the log 2 fold changes of DEGs in CD8^+^ TRM from RRMM versus NBM (*y*‐axis) and MM versus NBM (*x*‐axis) comparisons. The DEGs in both comparisons are highlighted. C) TSNE visualization of CD8^+^ TRM, color‐coded by *KLRB1* expression (left), and a bar chart comparing the expression levels of *KLRB1* across NBM and each MM stage (right). Significance was calculated with one way ANOVA. ****p < 0.0001. D,E) GSEA performed using gene sets associated with resident score, E) exhaustion score, and D) MSigDB (Hallmark, Reactcome, c5). Red represents upregulation in *KLRB1* ^high^ CD8^+^TRM, while blue indicates downregulation in *KLRB1* ^high^ CD8^+^ TRM. F) Expression levels of *KLRB1* and immune checkpoint genes in CD8^+^ TRM across NBM and three MM stages. Color intensity reflects gene expression levels. G) Pirate plot shows mRNA expression levels of *CLEC2D* and immune checkpoint ligand genes in the MMRF‐CoMMpass patient cohort. Comparation of *CLEC2D* and immune checkpoint ligand genes expression are displayed. Data were presented as mean ± SD. Significance was calculated with Student's *t*‐test. ****p < 0.0001. H) The mRNA expression levels of *CLEC2D* and immune checkpoint ligand genes in MM cell lines (*n* = 34) from the Human Protein Atlas database. I) Protein expression levels of CLEC2D in MM cell lines, with isotype control antibody staining shown in gray. Isotype antibody staining of U266 is used as a negative control. J) GO analysis of *CLEC2D* high versus low expression group up (top) and down (bottom) regulation genes based on MMRF‐CoMMpass cohort. In each of the GO domains— biological process (BP), cellular component(CC), and molecular function (MF)—we selected the top three pathways with the smallest *p*.adj values (*p*.adj < 0.05). K) Kaplan–Meier curves showing the impact of high versus low expression of *KLRB1* (GSE136337) and *CLEC2D* (MMRF‐CoMMpass cohort) on OS. *p*‐values were calculated using the log‐rank test. TRM, tissue resident memory; DEG, differentially expressed gene; GSEA, gene set enrichment analysis; NES, normalized enrichment score; MFI, mean fluorescence intensity; OS, overall survival; GO, gene ontology; CC, cellular component; MF, molecular function; and BP, biological process.

Although BM CD8⁺ TRM cells exhibit increased expression of NK‐related genes that positively correlate with cytotoxicity signatures (Figure 1F), we hypothesize that this transcriptional state reflects a dysfunctional or exhausted phenotype rather than effective antimyeloma activity. To explore this, we analyzed the expression levels of NK signature genes across NBM and the three progressive stages of MM (Figure S2A, Supporting Information). A distinct cluster of genes showed a stepwise increase as the disease progressed (i.e., increased from MGUS/SMM to MM and further enhanced in RRMM) (**Figure** **2**A). Furthermore, to identify shared co‐upregulated genes between RRMM and MM compared to NBM, we performed a differential gene expression analysis of CD8^+^ TRM from both conditions. This analysis revealed 18 genes that were significantly upregulated in CD8^+^ TRM from both RRMM and MM compared to NBM (Figure 2B; Table S2, Supporting Information). Among these, we selected *KLRB1* for further investigation due to two key reasons: 1) *KLRB1* encodes the inhibitory receptor CD161, representing a promising therapeutic target; and 2) its expression was notably higher in RRMM, indicating a potentially critical role in advanced stages of MM (Figure 2C).

To determine the function of CD161 in CD8^+^ TRM in MM, we categorized CD8^+^ TRM into *KLRB1* ^high^ and *KLRB1* ^low^ subsets. The gene set enrichment analysis (GSEA) revealed that *KLRB1* ^high^ cells were associated with exhaustion, migration, apoptosis, and hypoxia, whereas *KLRB1* ^low^ ones exhibited enhanced tissue residency and effector functions (Figure 2D,E). Furthermore, we compared *KLRB1* expression with that of other inhibitory receptor genes. Notably, distinct from *KLRB1*, most other inhibitory receptor genes (e.g., *PDCD1*, *CTLA4*, *LAG3*, and *TIGIT*) did not exhibit a disease‐stage‐dependent expression pattern (Figure 2F).

Given the potential role of *KLRB1* in suppressing CD8^+^ TRM function, we next investigated the expression of *KLRB1* ligand, *CLEC2D* in MM cells. First, we profiled the expression of several immunosuppressive receptor ligand genes, including *CLEC2D*, in CD138^+^ tumor cells from MM patients using bulk RNA‐seq data (MMRF‐CoMMpass cohort). Notably, *CLEC2D* expression was significantly higher than that of other immunosuppressive receptor ligand genes (e.g., *CD274* for PD‐1, *CEACAM1*/*LGALS9* for TIM3, *CD80*/*CD86* for CTLA‐4) (Figure 2G). Additionally, leveraging publicly available RNA‐seq data from 34 MM cell lines, we confirmed the expression of *CLEC2D* across multiple MM cell lines (Figure 2H). This finding was further validated at the protein level (Figure 2I). Next, we categorized patients into *CLEC2D* ^high^ and *CLEC2D* ^low^ expression groups and conducted a differential expression analysis based on the MMRF‐CoMMpass cohort. The gene ontology (GO) enrichment analysis of *CLEC2D* ^high^ versus *CLEC2D* ^low^ MM cells revealed an upregulation of biological processes (BP) and cellular components (CC) related to ribosome biogenesis, oxidative phosphorylation, and active transmembrane transport. Conversely, downregulated genes were enriched in pathways related to DNA damage repair and epigenetic regulation (Figure 2J; Table S3, Supporting Information). These adaptations may allow for the survival and proliferation of MM cells in a challenging BM‐TME.

Finally, we assessed the prognostic value of *KLRB1* and *CLEC2D* expression levels using two MM patient cohorts (GSE136337 and MMRF‐CoMMpass). Indeed, MM patients with high expression levels of *KLRB1* or *CLEC2D* had unfavorable overall survival (OS) (Figure 2K). In contrast, *CD274* (PD‐L1) and *CEACAM1* displayed a protective trend, with higher expression levels correlating with improved OS (Figure S2C, Supporting Information). The expression of other inhibitor receptors or ligands was not associated with clinical outcomes in MM patients (Figure S2B,C, Supporting Information).

### 2.3 CD161–CLEC2D Axis Represents a Major Inhibitory Pathway in CD8^+^ BM‐TRM in MM

**Figure 3:**
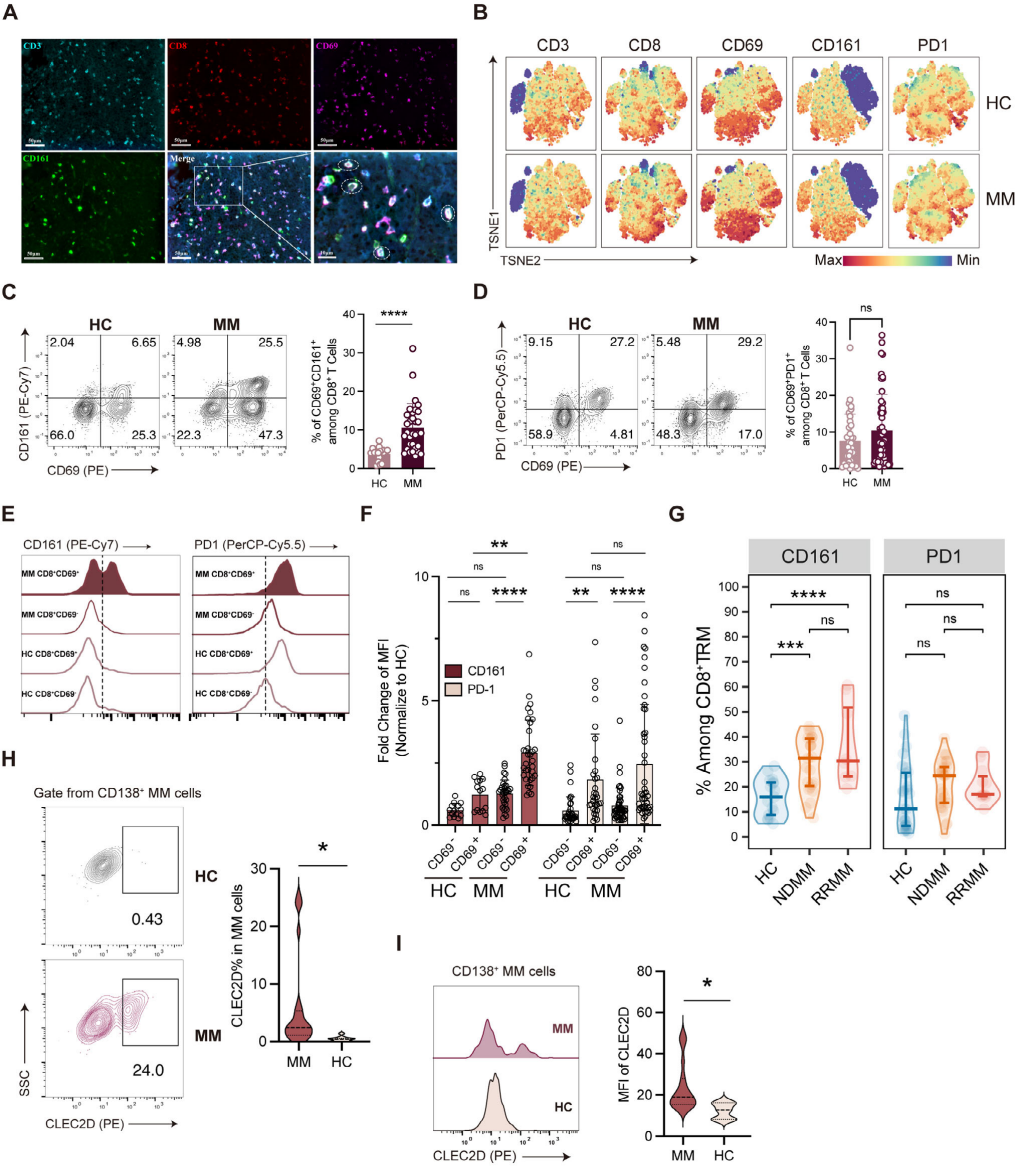
CD161–CLEC2D axis represents a major inhibitory pathway in CD8^+^ BM‐TRM in MM. A) Multiplex immunofluorescence staining for CD3 (cyan), CD8 (red), CD69 (purple), CD161 (green), and DAPI (blue). Scale bar: 50 and 10 µm. Dashed white circles enclose CD3^+^CD8^+^CD69^+^CD161^+^ cells in the MM BM biopsy sample. B) Representative TSNE plots depicting T cells from BM samples of HC and MM patients. C,D) Flow cytometric analysis of CD69 (*x*‐axis), C) CD161, and D) PD1 on BM CD8^+^ T cells between HC and MM patients (left). Comparison of CD69^+^PD1^+^ % and CD69^+^CD161^+^ % among CD8^+^ T cells between HC (*n* = 30) and MM (*n* = 50) (right). Data were presented as mean ± SD. Significance was calculated with Student's *t*‐test. ****p < 0.0001 and ns, no significance. E) Representative MFI curve for CD161 (left) or PD1 (right) on BM CD8^+^ T cells (classified as CD69^−^ or CD69^+^) from HC and MM. F) Fold change of MFI for CD161 and PD1 on BM CD8^+^ T cells (classified as CD69^−^ or CD69^+^) from HC (*n* = 30) and MM (*n* = 50) was analyzed by flow cytometry (normalized to HC CD69^‐^CD8^+^ T cells). Data were presented as mean ± SD. Significance was calculated with one way ANOVA. **p < 0.01, ****p < 0.0001 and ns, no significance. G) Distribution of CD161% (left) or PD1% (right) among CD8^+^ TRM for HC, NDMM and RRMM patients (HC, *n* = 17; NDMM, *n* = 23; and RRMM, *n* = 8). Data were presented as mean ± SD. Significance was calculated with one way ANOVA. ***p < 0.001, ****p < 0.0001, and ns, no significance. H) CLEC2D expression frequency and I) MFI in plasma cells from HC (*n* = 10) and tumor cells from MM patients (*n* = 20) were evaluated by flow cytometry. Data were presented as mean ± SD. Significance was calculated with Student's *t*‐test. *p < 0.05. NDMM, newly diagnosed multiple myeloma.

Building on the observation that *CLEC2D* expression in MM cells may contribute to immune evasion via its interaction with *KLRB1* (CD161) on CD8^+^ TRM, we next aimed to confirm these bioinformatics findings. First, we validated the expression of CD161 using immunofluorescence and flow cytometry. Multiplex immunofluorescence labeling of BM biopsy tissues from MM patients confirmed the co‐localization of CD3^+^, CD8^+^, CD69^+^, and CD161^+^ cells within the BM‐TME (**Figure** **3**A; Figure S3A, Supporting Information). Flow cytometry further revealed three prominent features associated with CD161 expression in BM: 1) MM CD8⁺ T cells and NK cells showed significantly increased CD161 expression versus HC, but no difference in CD4^+^ T cells (Figure S3C, Supporting Information); 2) among CD8^+^ T cells, the majority of CD161 expression was restricted to CD69^+^ TRM, but not CD69^‐^ Tcir (Figure 3B,C; Figure S3B,C, Supporting Information); 3) compared with HC, the expression of CD161 was significantly upregulated on CD8^+^ TRM isolated from MM patients (Figure 3C,E,F).

In contrast to CD161, no significant differences in other co‐inhibitory receptors between HC and MM BM CD8^+^ TRM were identified, including PD‐1 (Figure 3B,D–F), TIM3, and LAG3 (Figure S3D,E, Supporting Information). Notably, the proportion of CD161^+^ TRM in MM was substantially larger than that of PD‐1^+^ TRM (Figure S3F, Supporting Information). These findings may help explain the limited clinical efficacy of anti‐PD‐1, anti‐TIM3, or anti‐LAG3 therapies in MM.^[^ ^4^ ^]^

Finally, we would like to highlight additional features associated with the expression of CD161–CLEC2D in MM patients: 1) CD161, but not PD‐1 expression correlated with MM disease classification. We observed a significant increase in the proportion of CD161‐expressing CD8^+^ TRM in RRMM patients, whereas PD‐1 levels showed no notable differences (Figure 3G). 2) The expression of CD161 was age and gender independent in MM patients (Figure S3G, Supporting Information). 3) The major population of CD161^+^ BM CD8^+^ T cells was conventional T cells, not mucosal‐associated invariant T cells (MAIT cells) (Figure S3H,I, Supporting Information). 4) CLEC2D expression was highest in BM B cells from MM patients, followed by plasma cells and monocytes (Figure S3J–L, Supporting Information). Importantly, CLEC2D expression in plasma cells from MM patients was significantly higher than in those from HC (Figure 3H,I).

Overall, these findings highlight the CD161–CLEC2D axis as a potentially major immunosuppressive signaling pathway mediating CD8^+^ BM‐TRM dysfunction in MM.

### 2.4 CD161 Blockade Enhances CD8^+^ TRM Function Ex Vivo

**Figure 4:**
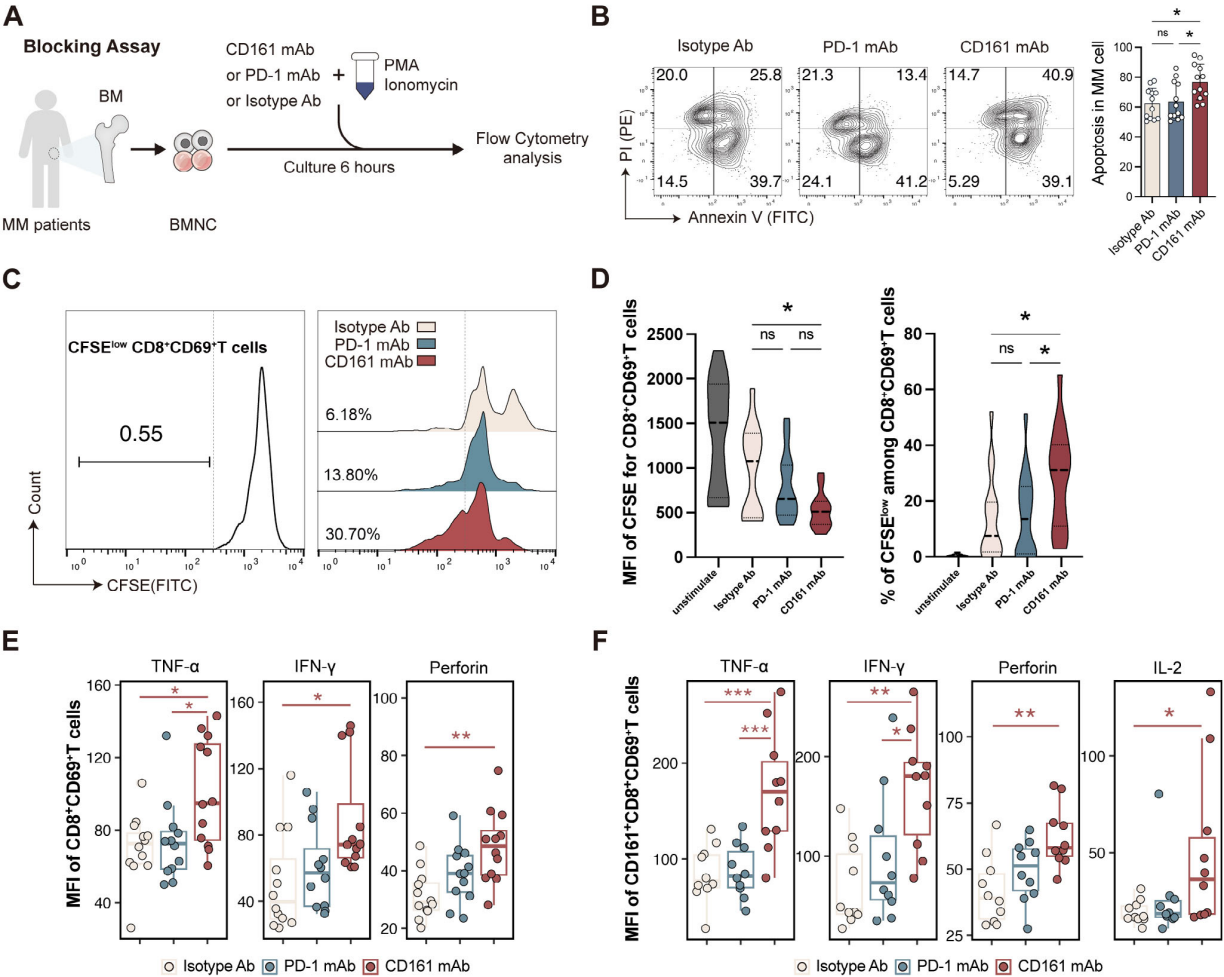
CD161 blockade enhances CD8^+^ TRM function ex vivo. A) Schematic of the experimental design. B) Flow cytometry analysis of apoptosis% in MM cells (CD138^+^) for three groups treated with isotype (*n* = 12), PD1 mAb (*n* = 12), and CD161 mAb (*n* = 12). Data were presented as mean ± SD. Significance was calculated with one way ANOVA. *p < 0.05 and ns, no significance. C) The gray curve shows the CFSE MFI of CD8^+^ CD69^+^T cells prior to culture, serving as negative control (left). The proliferation percentage of CD8^+^ CD69^+^T cells in each group is indicated (right). D) Comparison of CFSE MFI and CFSE^low^ % among CD8^+^ CD69^+^T cells across unstimulated (CD8^+^ CD69^+^T cells prior to culture), isotype (*n* = 17), PD1 mAbs (*n* = 17), and CD161 mAbs (*n* = 17). Data were presented as mean ± SD. Significance was calculated with one way ANOVA. *p < 0.05 and ns, no significance. E,F) The box plots show the expression of TNF‐α, IFN‐γ, Perforin, and IL‐2 after treated with isotype, PD1 mAbs, and E) CD161 mAbs in CD8^+^ CD69^+^T cells (*n* = 12), and F) CD161^+^ CD8^+^ CD69^+^T cells (*n* = 10). Data were presented as mean ± SD. Significance was calculated with one way ANOVA. *p < 0.05, **p < 0.01, and ***p < 0.001. BMNC, bone marrow mononuclear cell; PMA, phorbol‐12‐myristate‐13‐acetate; and CFSE, carboxyfluorescein succinimidyl ester.

Next, we interrogated the function of the CD161–CLEC2D pathway through an ex vivo blocking assay using primary bone marrow mononuclear cells (BMNCs) from MM patients. BMNCs were stimulated with phorbol‐12‐myristate‐13‐acetate (PMA) and ionomycin in the presence of anti‐CD161 monoclonal antibody (mAb), anti‐PD‐1 mAb, or Immunoglobulin G (IgG) isotype control for 6 h (**Figure** **4**A). Compared to the isotype control and PD‐1 mAb group, blocking CD161 significantly enhanced cell‐killing capacity against malignant plasma cells (CD138^+^ cells) (Figure 4B). To assess TRM proliferation, BMNCs were labeled with carboxyfluorescein succinimidyl ester (CFSE), and flow cytometry was used to measure CFSE dilution CD8^+^CD69^+^ cells. CD161 blockade promoted the proliferation of CD8^+^CD69^+^ cells (Figure 4C,D).

Next, cytokine production was evaluated. Anti‐CD161 treatment resulted in a notable increase in the proportions of Tumor Necrosis Factor‐α (TNF‐α)^+^, Interferon‐γ (IFN‐γ)^+^, and perforin^+^ cells within CD8^+^ T cells compared to the isotype control and PD‐1 blockade groups (Figure S4A,B, Supporting Information). When zoomed in to focus on CD8^+^CD69^+^ and CD161^+^CD8^+^CD69^+^ subsets, both the proportion of cytokine‐positive cells and their mean fluorescence intensity (MFI) levels showed significant increases upon CD161 blockade, suggesting enhanced activation and functional capacity of these specific subpopulations (Figure 4E,F; Figure S4C–F, Supporting Information). Importantly, IFN‐γ upregulation was the most pronounced, particularly in CD161^+^CD8^+^CD69^+^ subsets (Figure 4F; Figure S4E,F, Supporting Information). Additionally, Interleukin 2 (IL‐2) expression was detected in CD161^+^CD8^+^CD69^+^ cells, and its levels also showed a significant increase following CD161 blockade, further emphasizing the functional reactivation of this subset (Figure 4F; Figure S4E,F, Supporting Information). Together, CD161 suppresses the function of BM CD8^+^ T cells in freshly isolated samples from human patients.

### 2.5 NK1.1 Blockade Enhances CD8^+^ TRM Function and Extends Survival In Vivo

**Figure 5:**
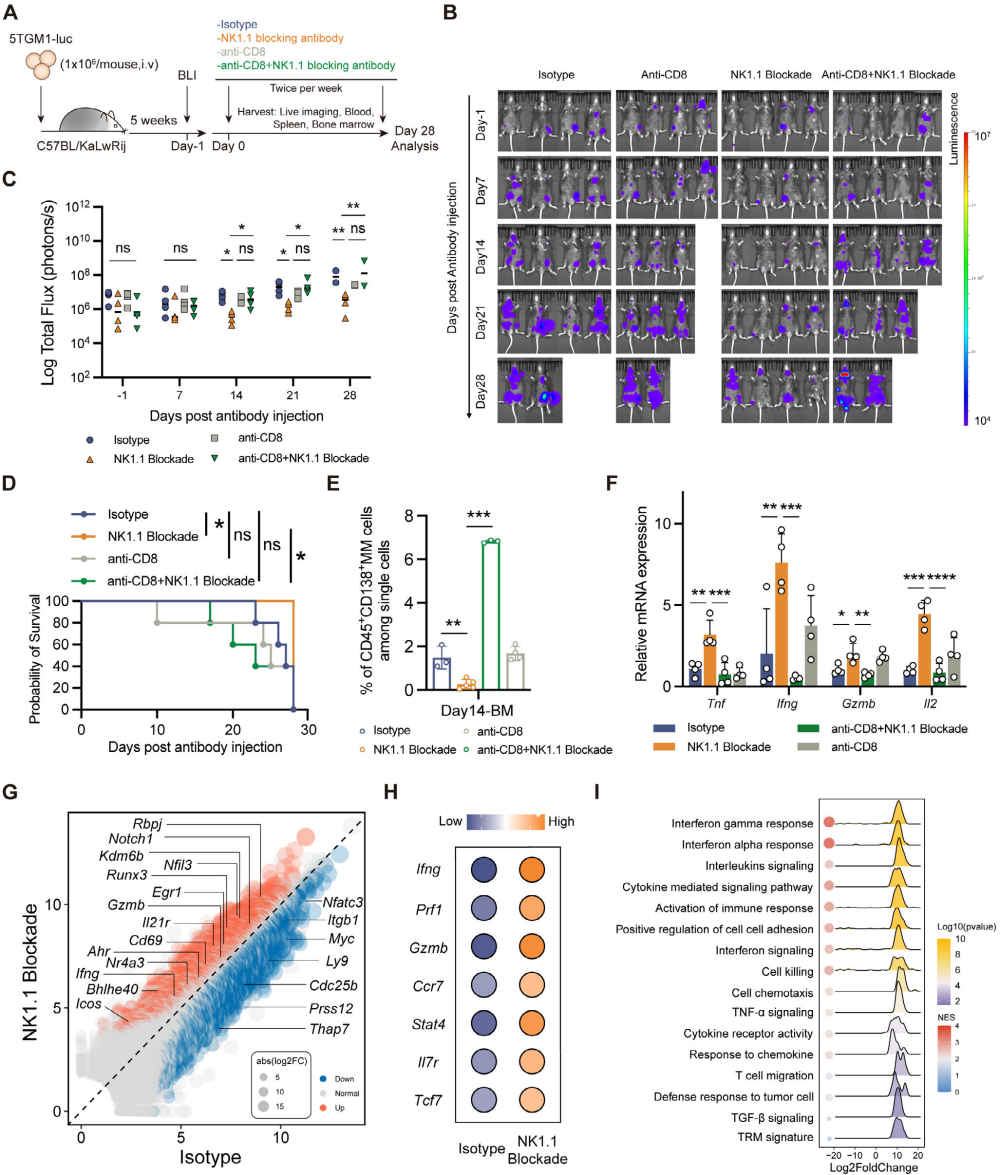
NK1.1 blockade enhances CD8^+^ TRM function and extends survival in vivo. A) Experimental design. B,C) Disease progression after antibody administration was quantified by B) bioluminescence imaging, with C) total flux quantified (*n* = 16). Data were presented as mean ± SD. Significance was calculated with one way ANOVA. *p < 0.05, **p < 0.01, and ns, no significance. D) Kaplan–Meier survival curve of MM‐bearing mice (*n* = 16) for four groups as described in panel (A) (starting from antibody injection). *p*‐values were calculated using the log‐rank test. *p < 0.05 and ns, no significance. E) Flow cytometry analysis of MM cells% (CD45^+^CD138^+^) in BM (*n* = 15), on day 14 post antibody injection. Data were presented as mean ± SD. Significance was calculated with one way ANOVA. **p < 0.01 and ***p < 0.001. F) Relative mRNA expression levels of *Tnf*, *Ifng*, *Gzmb*, and *Il2* in BM (*n* = 16) from four groups were compared at day 28 after antibody treatment by qPCR. Data were presented as mean ± SD. Significance was calculated with one way ANOVA. *p < 0.05, **p < 0.01, ***p < 0.001, and ****p < 0.0001. G) Differential expression gene analysis between NK1.1 blockade (*n* = 2) and isotype (*n* = 2) in BM CD8^+^ T cells of MM mice (day 14 after antibody administration). H) Bubble plot displaying expression levels of effector and stemness genes in two groups (NK1.1 blockade and isotype). I) Gene set enrichment analysis of DEGs (NK1.1 blockade versus isotype) was performed using MSigDB gene sets (Hallmark, Reactcome, c5). BLI, bioluminescence imaging; qPCR, real‐time fluorescence quantitative polymerase chain reaction.

To confirm these ex vivo findings, 5TGM1‐Luc cells were engrafted via tail vein injection into C57BL/KaLwRij mice, establishing a classical MM model within 5 weeks (**Figure** **5**A). By week 5, MM mice exhibited hind leg fractures and a significant increase in serum IgG2b levels (Figure S5A, Supporting Information). Examining mice spleen stained with Swiss dye under a light microscope further confirmed the presence of typical MM plasma cell morphology (Figure S5B, Supporting Information). Bioluminescence imaging (BLI) was initiated on day 1 relative to antibody treatment to assign mice with comparable BLI signals into four experimental groups. To determine whether the therapeutic effects of NK1.1 blockade were CD8^+^ T‐cell dependent, we performed CD8^+^ T cell depleted on two groups of mice (Figure 5A). MM mice were treated with NK1.1 mAb, IgG isotype control, CD8‐deplete mAb, CD8‐deplete mAb plus NK1.1 mAb, administered twice weekly. Tumor burden was monitored using in vivo BLI. Anti‐CD8 antibody treatment significantly reduced the percentage of CD8^+^ T cells in vivo (Figure S5C, Supporting Information).

NK1.1 blockade significantly reduced tumor burden on days 14, 21, and 28 post treatment (Figure 5B,C; Figure S5D, Supporting Information), and extended the survival of MM‐bearing mice (Figure 5D). BLI demonstrated that the combination of anti‐CD8 with NK1.1 blockade completely abolished both the tumor control and survival benefits observed with NK1.1 blockade monotherapy (Figure 5B–D). On days 7 and 14, NK1.1 blocking significantly decreased the proliferation of tumor cells, as evidenced by a reduction in KI67 expression (Figure S5E, Supporting Information). Moreover, by day 14 post‐treatment, NK1.1 blockade significantly reduced BM tumor burden compared to the isotype group. Still, the antitumor benefit conferred by NK1.1 blockade was completely abrogated​ when CD8⁺ T cells were simultaneously depleted (Figure 5E). Interestingly, CD8⁺ T‐cell depletion alone did not alter tumor burden relative to the isotype control, indicating that endogenous CD8⁺ T cells may be functionally exhausted and thus insufficient to mediate effective tumor control (Figure 5E).

To profile the impact of NK1.1 blocking on the tumor and immune compartments, PB was collected on day 3 post treatment, while spleen and BM samples were obtained on days 7 and 14 post‐treatment for flow cytometric analysis. The proportions of CD3^+^CD8^+^ cells in PB and BM, and CD8^+^CD69^+^ TRM in the BM were markedly increased in the NK1.1 blockade group (Figure S5F–H, Supporting Information). Consistent with the functional restoration observed in MM patient samples ex vivo (Figure 4E,F), NK1.1 blockade significantly boosted in vivo production of *Tnf*, *Ifng*, *Gzmb*, and *Il2* in mice, but this enhancement was substantially attenuated when NK1.1 blockade was combined with CD8^+^ T‐cell depletion (Figure 5F). On day 28, all mice were euthanized, and spleens were weighted. NK1.1 blockade significantly reduced spleen weight compared to the isotype control group (Figure S5I, Supporting Information). Additionally, the proportion of NK cells in the BM were reduced in the NK1.1‐treated group, although the overall number of NK cells remained low in both groups (Figure S5J, Supporting Information).

We next set out to investigate the transcriptional alterations driven by NK1.1 blockade in vivo, BM‐derived CD8^+^ T cells were sorted from the two groups (isotype and NK1.1 mAb) for bulk RNA‐seq. Using differential expression analysis, we observed that the expressions of pro‐TRM genes, including *Cd69*, *Bhlhe40*, *Nr4a3*, *Nothc1*, *Ahr*, and *Runx3*, were significantly elevated on CD8^+^ T cells from NK1.1 blockade‐treated mice, while genes facilitate T‐cell circulation, such as *Itgb1*, *Ly9*, *Cdc25b*, and *Prss12*, were downregulated (Figure 5G; Table S4, Supporting Information). Furthermore, NK1.1 blockade led to upregulation of effector‐cell genes (*Ifng*, *Prf1*, and *Gzmb*) and stemness‐like genes (*Ccr7*, *Stat4*, *Il7r*, and *Tcf7*) (Figure 5H). As shown in GSEA, NK1.1 blockade significantly activated the interferon pathway in CD8^+^ T cells, enhanced tissue residency, promoted proliferation, increased cytokine and chemokine secretion, and elevated their tumor cell‐killing capacity (Figure 5I; Figure S5K, Supporting Information). Furthermore, re‐analysis of single‐cell RNA‐seq data from a separate study (GSE220200)^[^ ^9^ ^]^ confirmed that CD161 blockade increased the TRM signature scores of T cells, particularly CD8^+^ T cells (Figure S5L, Supporting Information). Thus, CD161 delivers a key inhibitory signal to BM CD8^+^ TRM during anti‐MM responses in vivo.

### 2.6 CD161 Blockade Enhances CAR‐T Cytotoxicity, Reduces exhaustion, and Sustains a Resident Memory Signature

**Figure 6:**
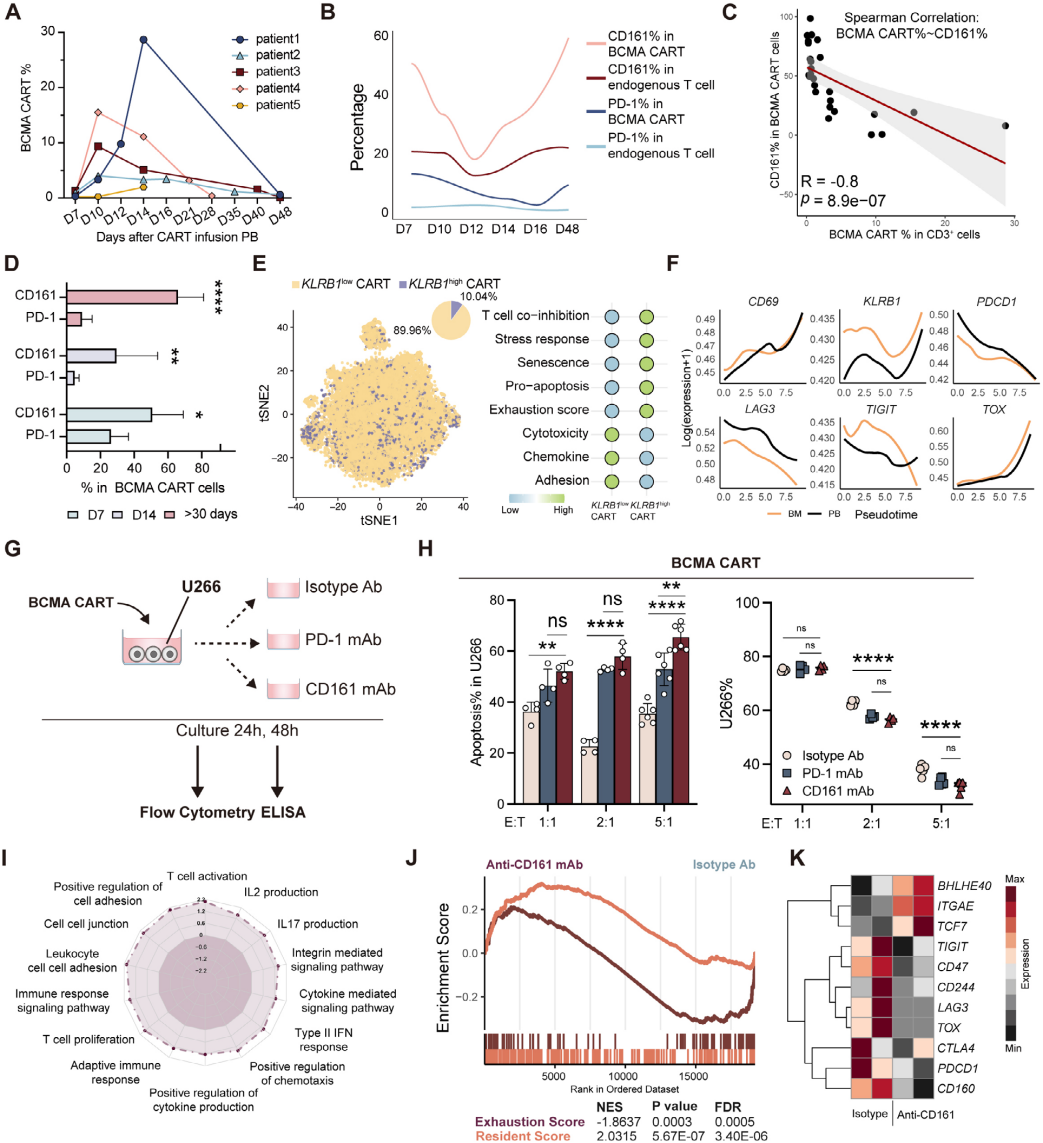
Dynamic regulation of CD161 during late‐stage CAR‐T‐cell expansion drives exhaustion and impacts antitumor efficacy. A) Dynamic changes of CART% in MM patients (*n* = 5) receiving BCMA CART at the indicated time points by flow cytometry. B) A fitted curve depicts the proportions of CD161 and PD1 expression in CART or T cells at the indicated time points post BCMA CART infusion. C) Spearman correlation analysis of CD161% in BCMA CART cells (*y‐*axis) and BCMA CART% (*x*‐axis) in MM patients (*n* = 24). Significance was calculated with the Spearman test. D) Comparison of CD161% and PD1% among BCMA CART cells on day 7 (*n* = 4), day 14 (*n* = 4), and >30 days (*n* = 7). Data were presented as mean ± SD. Significance was calculated with the Student's *t*‐test. *p < 0.05, **p < 0.01, and ****p < 0.0001. E) TSNE plot and pie chart (left) illustrating the distribution and proportion of high and low *KLRB1* populations in BCMA CART cells. Bubble plot (right) displaying the expression of eight curated gene signatures in these populations. F) Average expression of selected genes along the pseudotime trajectories for BCMA CART cells from BM and PB. G) Experimental design. H) Comparison of U266 cells% and apoptosis% in U266 across three treatment groups (isotype, CD161 mAb, and PD‐1 mAb) for BCMA CART cells (*n* = 4) after 48 h culture at different E:T (1:1, 2:1, and 5:1) ratios. Data were presented as mean ± SD. Significance was calculated with one way ANOVA. **p < 0.01, ****p < 0.0001, and ns, no significance. I,J) Gene set enrichment analysis of BCMA CART DEGs (anti‐CD161 versus isotype) using gene sets for resident score, J) exhaustion score, and I) MSigDB (Hallmark, Reactcome, and c5). K) Heatmap displaying expression levels of genes of BCMA CART between CD161 mAb and isotype Ab group. CART, chimeric antigen receptor t; BCMA, B cell maturation antigen; ELISA, enzyme‐linked immunosorbent assays.

While CAR‐T‐cell therapy has become an essential treatment for RRMM, addressing CAR‐T‐cell exhaustion remains a significant challenge.^[^ ^2^, ^3^ ^]^ Building on our findings that the CD161–CLEC2D interaction induces CD8^+^ BM‐TRM dysfunction and promotes MM progression, we next determined whether CD161 is expressed on CAR‐T cells in RRMM patients and whether it contributes to their functional exhaustion. First, we collected PB from five RRMM patients who underwent B‐cell maturation antigen (BCMA)‐directed CAR‐T therapy at multiple time points (Table S5, Supporting Information). Most patients exhibited a peak in CAR‐T‐cell expansion between days 10 and 14 post infusion (**Figure** **6**A; Figure S6A, Supporting Information). The expression of CD161 exhibited a biphasic pattern, with high expression in early BCMA CAR‐T cells after infusion, a decline reaching a nadir around days 10–14, which coincided with the peak of CAR‐T expansion, and a subsequent increase during CAR‐T contraction (Figure 6B; gating strategy in Figure S6B in the Supporting Information). The proportion of BCMA CAR‐T cells in CD3^+^ cells was significantly inversely correlated with the expression level of CD161 on BCMA CAR‐T cells (Figure 6C; Figure S6C, Supporting Information). Moreover, CD161 expression in BCMA CAR‐T cells was considerably higher than that in endogenous T cells, while PD‐1 expression showed no significant fluctuation (Figure 6B), and there was no correlation between PD‐1 levels and BCMA CAR‐T frequency (Figure S6C, Supporting Information). Critically, the proportion of CD161^+^ BCMA CAR‐T cells in MM was substantially larger than that of PD‐1^+^ BCMA CAR‐T cells, with the difference becoming even more pronounced at later stages (>30 days) (Figure 6D).

Next, we sorted the CAR‐T cells from PB and BM of one RRMM patient at day 35 after BCMA CAR‐T infusion for scRNA‐seq. Indeed, *KLRB1* expression was observed in CAR‐T cells from both the BM and PB (Figure 6E; Figure S6D, Supporting Information). CAR‐T cells were categorized into *KLRB1* ^high^ and *KLRB1* ^low^ subsets for subsequent analysis. *KLRB1* ^high^ CAR‐T cells displayed a higher expression of inhibitory genes (*PDCD1*, *LAG3*, *CTLA4*, and *TOX*), and cell migration‐related genes (*KLF2* and *S1PR1*) (Figure S6E). In contrast, *KLRB1* ^low^ CAR‐T cells highly expressed cytotoxicity genes (*GZMB*, *TCF7*, and *GNLY*), resident genes (*ITGAE*, *ITGA1*, *CXCR3*, *RUNX3*, and *PRMD1*) (Figure S6E, Supporting Information). Functionally, *KLRB1* ^high^ CAR‐T cells were enriched with exhaustion, stress response, and apoptosis features, whereas *KLRB1* ^low^ CART cells exhibited enhanced cytotoxicity, chemokine production, and adhesion functions (Figure 6E). We further examined the expression dynamics of *CD69*, *KLRB1*, and several representative exhaustion‐related genes (*PDCD1*, *LAG3*, *TIGIT*, and *TOX*) in CAR‐T cells along pseudotime. Notably, we observed a progressive increase in *CD69* expression as pseudotime advanced (Figure 6F). *KLRB1* exhibited a biphasic pattern, with an initial decrease in expression followed by a subsequent increase (Figure 6F), consistent with the expression pattern of CD161 on BCMA CAR‐T cells (Figure 6B). *TOX* is a well‐established master regulator of T‐cell exhaustion.^[^ ^10^ ^]^ Along the pseudotime, the dramatic induction of *TOX* at the later stage correlated very well with *KLRB1* expression. In contrast, other inhibitory receptor genes exhibited an opposite expression pattern (Figure 6F). Notably, CAR‐T cells in the BM showed higher *KLRB1* expression compared to those in PB (Figure 6F), suggesting that *KLRB1* may exert a stronger inhibitory effect on CAR‐T cells within the BM–TME and a potential link between *KLRB1* and TRM programs. Together, these results strongly suggest that *KLRB1* is the major inhibitory receptor at the later stages of CAR‐T differentiation.

To probe the functional importance of CD161 in BCMA CAR‐T cells, we conducted in vitro culture assays. BCMA CAR‐T cells were cultured with U266 cells at varying effector‐to‐target (E: T) ratios (1:1, 2:1, 5:1) in the presence of anti‐CD161 mAb, anti‐PD‐1 mAb, or an IgG isotype control for 24 or 48 h (Figure 6G; Figure S6F, Supporting Information). As expected, CD161 blockade significantly promoted BCMA CAR‐T‐cell proliferation, increased IFN‐γ secretion (Figure S6G, Supporting Information), and enhanced killing compared to the anti‐PD‐1 and isotype groups (Figure 6H). Additionally, we validated this finding using a different CAR (GPRC5D CAR) (Figure S6F,H–J, Supporting Information). Next, BCMA CAR‐T cells treated with either CD161 blockade or the isotype, and activated by CD3/CD28 for 24 h were subjected to RNA‐seq. Differential expression analysis revealed that CD161 blockade enhanced the expression of pro‐TRM (*ITGAE*) and effector (*IL4R*) genes (Figure S6K and Table S6, Supporting Information). Functionally, CD161 blockade activated interferon signaling in CAR‐T cells (Figure S6L, Supporting Information), promoted their proliferation and activation, increased cytokine and chemokine secretion, and enhanced TRM characteristics (Figure 6I–K). Furthermore, transcriptional profiling revealed a marked reduction in exhaustion‐associated genes, including *PDCD1*, *LAG3*, *TOX*, and *TIGIT*, in CAR‐T cells treated with anti‐CD161 (Figure 6K). Together, *KLRB1* is a critical inhibitory receptor dampening CAR‐T function.

### 2.7 Identification of a *KLRB1*‐Driven Exhaustion Program in CD8^+^ TRM across Hematological Malignancies

**Figure 7:**
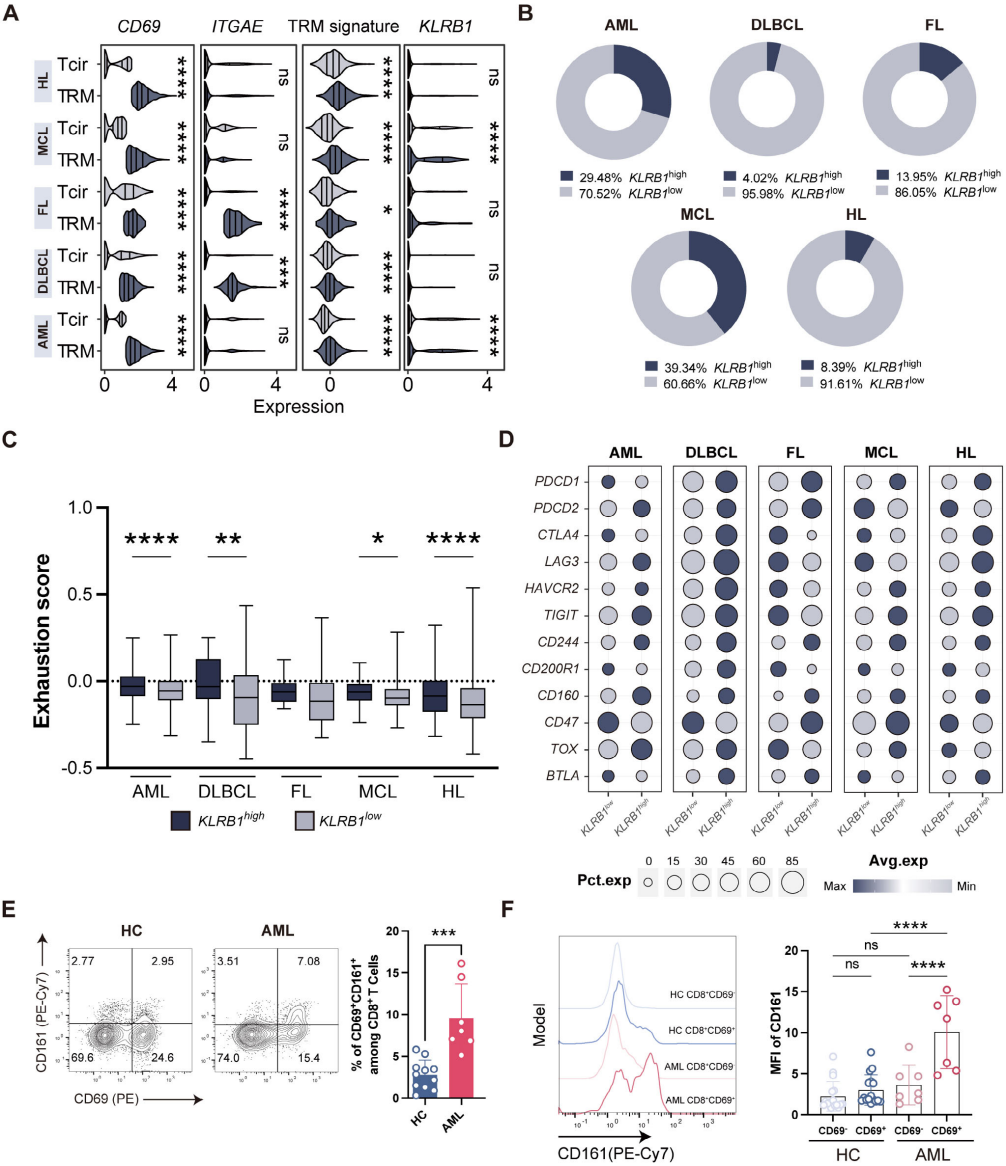
Identification of a *KLRB1*‐driven exhaustion program in CD8^+^ TRM across hematological malignancies. A) Stacking violin diagram depicting the expression levels of *CD69*, *ITGAE*, *KLRB1*, and TRM signature scores in TRM and Tcir across hematological malignances tissues. Data were presented as mean ± SD. Significance was calculated with the Student's *t*‐test. *p < 0.05, ***p < 0.001, ****p < 0.0001, and ns, no significance. B) Ring pie charts displaying the proportion of high and low *KLRB1* populations among CD8^+^ TRM in hematological malignances. C) Boxplot comparing exhaustion scores between high and low *KLRB1* expressing CD8^+^ TRM in hematological malignances. Data were presented as mean ± SD. Significance was calculated with the Student's *t*‐test. *p < 0.05, **p < 0.01, and ****p < 0.0001. D) Bubble plot showing the expression of representative exhaustion genes between *KLRB1* high and low cell populations in CD8^+^ TRM. E) Flow cytometric analysis of CD69 (*x*‐axis), CD161 (*y*‐axis) protein levels in BM CD8^+^ T cells from HC (*n* = 11) and AML patients (*n* = 7) (left). Comparison of CD69^+^CD161^+^% among CD8^+^ T cells between HC and AML (right). Data were presented as mean ± SD. Significance was calculated with Student's *t*‐test. ***p < 0.001. F) Representative MFI curve for CD161 on BM CD8^+^ T cells (classified as CD69^−^ or CD69^+^) from HC (*n* = 11) and AML (*n* = 7) (left). Comparison of MFI for CD161 on CD69^−^ and CD69^+^ BM CD8^+^T cells from HC and AML (right). Data were presented as mean ± SD. Significance was calculated with one way ANOVA. ****p < 0.0001 and ns, no significance. AML, acute myeloid leukemia; DLBCL, diffuse large B‐cell lymphoma; FL, follicular lymphoma; MCL, mantle cell lymphoma; HL, Hodgkin lymphoma.

To investigate whether the *KLRB1*‐driven exhaustion program is present in CD8^+^ TRM across other hematologic malignancies, we analyzed published scRNA‐seq datasets from patients with acute myeloid leukemia (AML), diffuse large B‐cell lymphoma (DLBCL), follicular lymphoma (FL), mantle cell lymphoma (MCL), Hodgkin lymphoma (HL) (Figure S7A, Supporting Information). CD8^+^ TRM and Tcir cells were identified as CD8^+^CD69^+^ and CD8^+^CD69^‐^ cells within the BM–TME, respectively (**Figure** **7**A). For DLBCL and FL, *ITGAE* was selected as an additional marker to define TRM (Figure 7A). Comparing the TRM signature scores between TRM and Tcir cells revealed that all identified TRM consistently exhibited higher TRM signature scores (Figure 7A). Moreover, CD8^+^ TRM demonstrated a strong correlation between cytotoxicity and exhaustion in TME across all malignancies (Figure S7B, Supporting Information). Notably, the expression of *KLRB1* was significantly upregulated in CD8^+^ TRM from AML and MCL (Figure 7A). To further explore whether *KLRB1* expression correlates with T‐cell exhaustion, CD8^+^ TRM were categorized into *KLRB1* ^high^ and *KLRB1* ^low^ subsets. Although the expression varied, *KLRB1^high^* subset could be detected in CD8^+^ TRM across all hematological malignancies (Figure 7B). Except for FL, *KLRB1* ^high^ CD8^+^ TRM consistently showed higher exhaustion scores (Figure 7C). Notably, in AML, *KLRB1* ^high^ CD8^+^ TRM exhibited elevated expression of multiple exhaustion‐related markers such as *TOX*, *LAG3*, *TIGIT*, and *CD160*, except *PDCD1*, whose expression pattern was opposite from that of *KLRB1* (Figure 7D). Flow cytometry analysis revealed that CD8^+^ TRM from AML patients exhibited significantly higher CD161 expression compared to HC (Figure 7E,F). In MCL, *KLRB1* ^high^ CD8^+^ TRM showed a similar trend with increased expression of exhaustion‐related genes (Figure 7D; Figure S7C, Supporting Information). In DLBCL and HL, *KLRB1* ^high^ CD8^+^ TRM also display higher levels of inhibitory markers, albeit with a less pronounced difference compared to AML and MCL (Figure 7D). Together, these findings suggest that *KLRB1* expression can be detected in CD8^+^ TRM infiltrating most hematological tumors. *KLRB1* expression, but not PD‐1 expression, correlates with TRM exhaustion, suggesting that *KLRB1* may deliver a broad inhibitory signal to CD8^+^ TRM in hematological malignancies.

**Figure 8:**
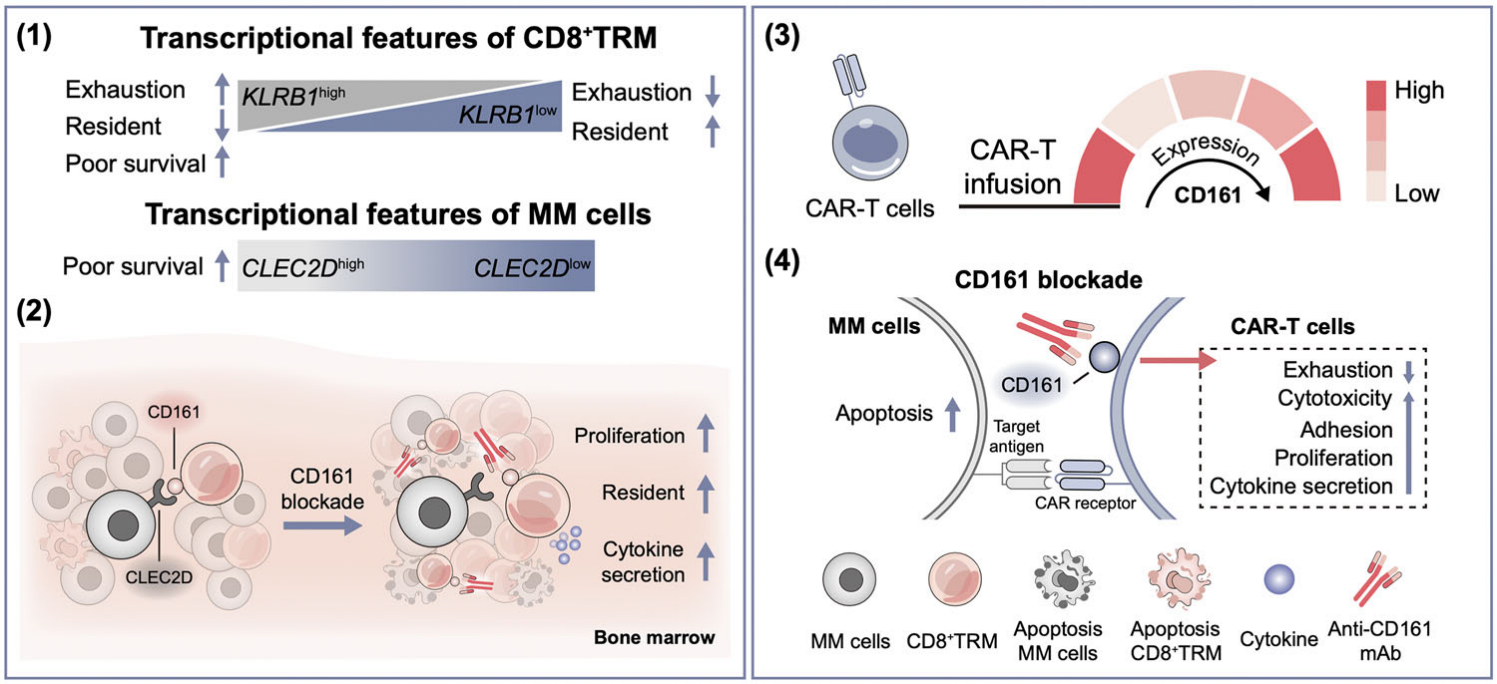
Schematic model of content of this study. CD161–CLEC2D axis marks dysfunctional CD8⁺ TRM state and poor prognosis in MM. Blocking CD161 enhances TRM function, including tissue residency, proliferation, and antitumor activity. Moreover, in RRMM patients, the expression of CD161 in CAR‐T exhibited a biphasic pattern. *KLRB1* is the major inhibitory receptor at the later stages of CAR‐T differentiation. CD161 blockade enhances CAR‐T cytotoxicity, reduces exhaustion, and sustains a resident memory signature.

## 3 Discussion

The advent of ICIs has revolutionized the treatment for many solid tumors. However, the clinical outcomes of PD‐1, LAG‐3, and CTLA‐4 targeting in MM are disappointing.^[^ ^4^ ^]^ This lack of efficacy emphasizes the unique challenges of MM's immunosuppressive TME and the urgent need to identify novel therapeutic targets.^[^ ^11^ ^]^ BM CD8^+^ TRM have garnered attention in MM. While these cells exhibit antimyeloma responses, they upregulate inhibitory receptors, such as PD‐1 and TIGIT, leading to functional impairment.^[^ ^6b^ ^]^ However, single‐agent blockade of these receptors is not effective to restore the TRM function. Our study identified *KLRB1* (CD161) as a novel inhibitory receptor controlling the immunosuppressive and dysfunctional state of CD8^+^ BM TRM in MM. CD161 interacts with CLEC2D, expressed on MM cells and other cells within the MM TME, positioning the CD161–CLEC2D axis as a key driver of immune suppression.

Additionally, our results show that blocking CD161 effectively alleviates exhaustion while promoting TRM‐like characteristics in CAR‐T cells, including enhanced tissue residency and persistence within the MM BM–TME. These improvements in residency and sustained antitumor functionality suggest that CD161 inhibition is a promising strategy for MM CAR‐T therapy. Intriguingly, *KLRB1* (CD161) expression in CAR‐T cells exhibits a biphasic pattern, reflecting a dynamic interplay between CAR‐T and the MM TME. We propose that CLEC2D in the microenvironment drives high CD161 expression in the early post‐infusion stage, partially inhibiting CAR‐T activity. However, at early stages, CD161 alone is insufficient to induce terminal exhaustion. Thus, CAR‐T continues to expand and target MM cells. At the peak of CAR‐T expansion, strong activation signals and reduced CLEC2D expression might lead to CD161 downregulation and enable efficient tumor cell killing. In the late post‐infusion phase, as tumor burden decreases, residual antigens induce the re‐expression of CD161, accelerating CAR‐T exhaustion and functional decline. This stage‐specific pattern highlights the potential to target CD161 to sustain CAR‐T activity and enhance residual tumor clearance. However, the precise regulatory mechanisms underlying this biphasic pattern require further investigation.

In addition to MM, our findings reveal a *KLRB1*‐driven exhaustion program in CD8^+^ TRM across various hematologic malignancies, a pattern also observed in solid tumors.^[^ ^8^, ^9^, ^12^ ^]^ Notably, in Human Papillomavirus (HPV)‐positive oropharyngeal squamous cell carcinoma, elevated CD161 expression on CD8^+^ TRM correlates with reduced antitumor activity, further supporting the relevance of TRM‐specific exhaustion mechanisms across tumor types.^[^ ^12b^ ^]^ Moreover, high CD161 expression has been reported to be associated with poor outcomes in several malignancies. For instance, it correlates with worse prognosis in hepatocellular carcinoma^[^ ^13^ ^]^ and shorter relapse‐free survival in AML patients 3 months post‐transplant.^[^ ^14^ ^]^ In MM, our transcriptomic analysis revealed that higher *KLRB1* and *CLEC2D* expressions correlate with worse survival. However, opposite trends have been observed in certain cancers, such as pancreatic ductal adenocarcinoma, where patients with low tumor‐infiltrating CD161^+^CD8^+^ T cells exhibited the worst survival.^[^ ^12a^ ^]^ This highlights a tumor‐type‐specific role of CD161, likely influenced by the distinct TME and the dynamic balance between activation and exhaustion of CD161^+^CD8^+^ T cells.

In summary, our study highlights the critical role of the CD161–CLEC2D axis in mediating TRM‐specific immunosuppression within the MM BM–TME, thereby facilitating disease progression. These findings demonstrate that the transition of CD8^+^ T cells into NK‐like T cells is closely linked to T‐cell dysfunction. Targeting the CD161–CLEC2D pathway emerges as a promising therapeutic strategy to mitigate immunosuppression and enhance the efficiency of immunotherapy against MM (**Figure** **8**).

## 4 Experimental Section

### Cell Lines

The murine MM cell line 5TGM1‐luc (RRID:CVCL_VI66) and the human MM cell lines ARP1 (RRID:CVCL_D523), H929 (RRID:CVCL_1600), RPMI 8226 (RRID:CVCL_0014), and U266 (RRID:CVCL_GZ72) were kindly provided by Zhou and co‐workers (Cancer Research Institute, School of Basic Medical Sciences, Central South University) and verified through short tandem repeat (STR) authentication. All cell lines were confirmed to be free from other microbial contamination. All cell lines were cultured in complete RPMI 1640 medium (supplemented with 10% fetal bovine serum and 1% penicillin–streptomycin). Cells were maintained at 37 °C in a humidified atmosphere with 5% CO_2_ and 21% O_2_, with the medium refreshed twice weekly.

### Collection of Human Samples

Fresh PB and BM aspirates from healthy donors, MM and AML patients at diagnosis, were collected at the Third Xiangya Hospital of Central South University. In total, 50 MM patients (with 50 BM and 20 PB samples), 7 AML patients (with 7 BM samples), and 30 HC (with 30 BM and 10 PB samples) were tested (Table S7, Supporting Information). PB mononuclear cells (PBMCs) and BMNCs were isolated using density gradient centrifugation with Ficoll (Sigma–Aldrich). The percentage of MM cells in BM samples was assessed using ten‐color single‐tube flow cytometry. For the generation of CAR‐T cells, fresh PBMCs were obtained by apheresis from patients. All donors and patients provided written informed consent, and the study was approved by the Ethical Committee of the Institute of the Third Xiangya Hospital of Central South University (Approval No. 24836).

### Experimental Mouse Models

Animal studies were conducted using female C57BL/KaLwRij (KAL) mice (aged 6–10 weeks), which were kindly provided by Zhou and co‐workers^[^ ^15^ ^]^ (Cancer Research Institute, School of Basic Medical Sciences, Central South University). All mice were housed under specific pathogen‐free conditions with unrestricted access to food and water. The study protocol (#CSU‐2024‐0169) was approved by the Department of Laboratory Animals at Central South University. All experiments involving mice were performed in strict compliance with ethical standards and guidelines for animal research.

### Flow Cytometry

To identify CAR‐T cells by flow cytometry, mononuclear cells isolated from PB or BM were prewashed and incubated with biotinylated human BCMA protein (ACROBiosystems) for 1 h on ice. After incubation, the cells were washed once with staining buffer. Expression of the anti‐BCMA CAR was assessed using PE (P‐phycoerythrin)‐conjugated streptavidin. Additional surface markers were stained for 30 min on ice using the following antibodies: APC (Allophycocyanin) antihuman CD3 (OKT3), BV510 antihuman CD45 (2D1), PE/Cyanine7 antihuman CD161 (W18070C), PerCP/Cyanine5.5 antihuman PD‐1 (NAT105), and LIVE/DEAD fixable viability dye. After staining, cells were washed and resuspended in PBS (Phosphate Buffered Saline) for further analysis. Other surface staining procedures involved incubating cells with antibodies on ice in the dark for 20 min, followed by washing with PBS and resuspension for analysis. For intracellular protein staining, cells were fixed and permeabilized using the Intracellular Fixation and Permeabilization Buffer kit (eBioscience) and subsequently stained with intracellular antibodies for 30 min. The following antibodies were used: PE antihuman Perforin (B‐D48), APC/Fire750 antihuman Granzyme B (QA16A02), FITC (Fluorescein Isothiocyanate) antihuman IFN‐γ (4S.B3), Pacific Blue antihuman TNF‐α (MAb11), APC antihuman IL‐2 (MQ1‐17H12), and Alexa Fluor 700 antimouse Ki67 (SolA15). All samples were analyzed using a Navios flow cytometer (Beckman Coulter) and FlowJo 10.8.1 software (see Figures S3B,J and S6B for gating strategies, Supporting Information). Detailed antibody information is provided in Table S8 (Supporting Information).

### Immunohistochemistry and Multiplex Immunofluorescence Staining

Tissue specimens were fixed in 4% formalin for 48 h and embedded in paraffin for sectioning. The sections were incubated overnight at 4 °C with primary antibodies against CD161 (1:900, ab302564, Abcam), followed by PBS washing. Staining was performed using an automated immunostainer (Leica Bond‐III, Dako Autostainer Link 48). Finally, section images were scanned with a NanoZoomer S360. Swiss staining was performed following standard procedures. For fluorescent multiplex immunohistochemistry (mIHC), a tyramide signal amplification (TSA)‐based fluorescence kit was used according to the manufacturer's protocol. The sections were incubated overnight at 4 °C with the following primary antibodies: anti‐CD161 (1:1000, ab302564, Abcam), anti‐CD3 (1:4000, ab237721, Abcam), anti‐CD8 (1:1000, CL488‐65135, Proteintech), anti‐CD69 (1:1000, ab233396, Abcam), and anti‐CD138 (1:1000, 67155‐1‐Lg, Proteintech). After completing the final TSA cycle, DAPI (4',6‐diamidino‐2‐phenylidole) was applied for counterstaining at a 1:1000 dilution for 10 min. Fluorescence microscopy images were acquired using the Pannoramic SCAN II system and visualized by CaseViewer (3D HISTECH).

### Ex Vivo Blocking Assays

Primary mononuclear cells isolated from the BM of MM patients were stimulated with PMA and ionomycin (eBioscience) and cultured with 5 µg mL^−1^ anti‐CD161 mAb (HP‐3G10), anti‐PD‐1 mAb (EH12.2H7), or mouse IgGκ isotype control Ab (MG1‐45) for 6 h.^[^ ^16^ ^]^

### Cell Apoptosis Experiment

After 6 h of culture, cells were stained with APC‐conjugated CD138 (DL‐101) and BV510‐conjugated CD45 (2D1), followed by staining with the FITC Annexin V/Dead Cell Apoptosis Kit (Invitrogen). Apoptosis (%) was calculated by determining the percentage of Annexin V^+^ cells among CD138^+^ tumor cells using flow cytometry.

### T‐Cell Proliferation Experiment

Primary cells were prestained with CellTrace CFSE dye (Invitrogen) prior to culture. The CFSE input before culture served as the baseline for unstimulated cells. After culture, cells were stained with APC‐conjugated CD3 (OKT3), APC‐Cy7‐conjugated CD8 (SK1), and PE‐conjugated CD69 (FN50). CFSE intensity post‐culture was measured by flow cytometry.

### Cytokine Secretion Detection

Cells were cultured with a mixture of PMA, ionomycin, and protein transport inhibitors (eBioscience) for 6 h and subsequently collected for staining with PerCP‐conjugated CD3 (SK7), APC‐Cy7‐conjugated CD8 (SK1), PE‐conjugated CD69 (FN50), PerCP‐conjugated CD69 (FN50), and PE‐Cy7‐conjugated CD161 (W18070C). Following fixation and permeabilization using the intracellular fixation and permeabilization buffer kit (eBioscience), intracellular staining was performed using the following antibodies: PE‐conjugated Perforin (B‐D48), APC/Fire750‐conjugated Granzyme B (QA16A02), FITC‐conjugated IFN‐γ (4S.B3), Pacific Blue‐conjugated TNF‐α (MAb11), and APC‐conjugated IL‐2 (MQ1‐17H12). Cytokine secretion levels in CD8^+^, CD8^+^CD69^+^, and CD8^+^CD69^+^CD161^+^ cells were analyzed by flow cytometry.

### Polymerase Chain Reaction with Reverse Transcription

PB samples were collected at multiple time points before and after CAR‐T‐cell infusion (Table S5, Supporting Information). CAR copy numbers in PB were quantified using the Detection Kit for Blood/Cell CAR Copy Number (Bioswan). TaqMan real‐time polymerase chain reaction (PCR) was performed on the SLAN (Sansure Real‐Time PCR Analysis)–YGP system (Sansure Biotech). Total RNA was extracted from PBMCs using Trizol reagent (Sangon Biotech). Reverse transcription was carried out according to the manufacturer's protocol using a reverse transcription kit. Relative transcript expression levels were quantified using AceQ quantitative PCR (qPCR) SYBR Green Master Mix (Vazyme) with a CFX touch real‐time PCR System (Bio‐Rad). The primers used in the study were as follows: *Ifng* (forward, 5′‐ATGAACGCTACACACTGCATC‐3′; reverse, 5′‐CCATCCTTTTGCCAGTTCCTC‐3′), *Tnf* (forward, 5′‐CCCTCACACTCAGATCATCTTCT‐3′; reverse, 5′‐GCTACGACGTGGGCTACAG‐3′), *Gzmb* (forward, 5′‐CCACTCTCGACCCTACATGG‐3′; reverse, 5′‐GGCCCCCAAAGTGACATTTATT‐3′), *Il2* (forward, 5′‐TGAGCAGGATGGAGAATTACAGG‐3′; reverse, 5′‐GTCCAAGTTCATCTTCTAGGCAC‐3′), and *Beta actin* (forward, 5′‐ATGTGGATCAGCAAGCAGGA‐3′; reverse, 5′‐AAGGGTGTAAAACGCAGCTCA‐3′).

### Culture of CAR‐T Cells with Tumor Cells

BCMA CAR‐T or GPRC5D CAR‐T cells were cultured in RPMI 1640 medium supplemented with 10% fetal bovine serum, 1% penicillin/streptomycin, and 50 ng mL^−1^ human IL‐2 (AbMole) at 37 °C, 5% CO_2_, and 21% O_2_. U266 cells were seeded into 96‐well plates at a density of 5 × 10^5^ cells per well. To assess the effect of anti‐CD161 mAb on CAR‐T cells, CAR‐T cells were cultured with U266 cells at varying effector‐to‐target (E:T) ratios (1:1, 2:1, and 5:1) in the presence of 5 µg mL^−1^ anti‐CD161 mAb (HP‐3G10), anti‐PD‐1 mAb (EH12.2H7), or mouse IgGκ isotype control antibody (MG1‐45) in 96‐well plates. After 24 or 48 h of culture, cells were collected and stained using the FITC Annexin V/Dead Cell Apoptosis Kit (Invitrogen). Tumor cells were gated based on side scatter (SSC) and forward scatter (FSC) by flow cytometry. The percentage of Annexin V^+^ cells among tumor cells was calculated to determine the apoptosis rate of U266 cells. After 48 h of culture, supernatants from 96‐well plates were collected, and IFN‐γ levels were measured using enzyme‐linked immunosorbent assays (ELISA) according to the manufacturer's protocol (Abbkine, Cat#KTE6011). Optical density (OD) was determined using a Synergy HTX microplate reader (141222F) set to 450 nm.

### CAR‐T‐Cell RNA Sequencing

CAR‐T cells were cultured in 96‐well plates in the presence of 5 µg mL^−1^ anti‐CD161 mAb (HP‐3G10) or mouse IgGκ isotype control antibody (MG1‐45). Stimulation was achieved using 5 µg mL^−1^ anti‐CD3 (BioLegend, Cat#317 326) and anti‐CD28 mAbs (BioLegend, Cat#302 934). After 24 h, cells were collected, and total RNA was extracted using the RNA rapid extraction kit (RNAfast200, Cat#220 011) for RNA sequencing.

### Mouse Experiments

The 5TGM1 MM model was established by intravenously injecting luciferase‐expressing 5TGM1 cells (1 × 10⁶ cells in 200 µL of PBS) into C57BL/KaLwRij mice. Disease progression was monitored by assessing tumor burden through BLI and measuring serum IgG_2b_ levels. Mouse IgG_2b_ in serum was quantified using the mouse IgG_2b_ ELISA Kit (#E99‐109, Bethyl Laboratories, USA). For BLI, mice were anesthetized using a precision isoflurane vaporizer (VetEquip, Livermore, CA, USA) with 4% isoflurane for 5 min, followed by intraperitoneal administration of luciferin (15 mg kg^−1^, Abiowell). Ten minutes post injection, images were captured using an IVIS Spectrum System with automatic exposure settings. BLI was performed weekly, and signal flux (photons s^−1^) was quantified using the Living Image software package (PerkinElmer). NK1.1 is the homologous molecule of human CD161 in mice. The NK1.1 mAb is similar to the antihuman CD161 mAb. On week 5, post‐tumor injection, mice exhibiting increased bioluminescence signals were randomized into two treatment cohorts: an isotype control antibody group (Cat#401411, BioLegend) and an anti‐NK1.1 mAb group (Cat#108759, BioLegend), with 9 mice per group. Both groups started with an approximately equivalent mean tumor burden. Antibodies were administered via intraperitoneal injection (5 mg kg^−1^) twice per week. Samples were collected at various time points for analysis. From days 3–28 post treatment, PB, spleen, and BM were harvested and processed into single‐cell suspensions. For flow cytometry, the suspensions were incubated with an Fc blocker (anti‐mouse CD16/CD32, eBioscience) and stained with surface and intracellular antibodies. Additionally, on day 14 post‐antibody administration, BM single‐cell suspensions were collected, and CD8a^+^ T cells were isolated using the CD8a^+^ T‐cell isolation kit (Miltenyi, Cat#130‐104‐075) following the manufacturer's protocol. Isolated CD8a^+^ T cells underwent RNA sequencing for further analysis. All surviving mice were euthanized on day 28. Detailed antibody information is provided in Table S8 (Supporting Information).

### CD8^+^ T‐Cell Depletion

To examine the role of the CD8^+^ T‐cell population in mediating NK1.1 mAb antitumor responses, after successfully establishing the MM mouse model, the mice were randomly assigned into four experimental groups (isotype group, NK1.1 blockade group, CD8 depletion group, and CD8 depletion with NK1.1 blockade group) based on BLI tumor burden data obtained during week 5. Groups started with an approximately equivalent mean tumor burden. The CD8^+^ T population in MM mice was depleted using antimouse CD8a antibody (Cat#100764, BioLegend, 10 mg kg^−1^). The antimouse CD8a antibodies were administered by intraperitoneal route 1 day before NK1.1 administered twice per week. The efficiency of CD8^+^ T‐cell depletion was confirmed with anti‐CD8a antibody (Cat#46‐0081‐80, eBioscience) staining by flow cytometric analysis on days 3, 5, 7, 14, and 21.

### Bulk RNA‐seq and Data Analysis

Transcriptome sequencing was performed on the DNBSEQ (DNA Nanoball sequencing) platform with paired‐end (PE150) cycles. Raw FASTQ files were trimmed and filtered by Fastp (version 0.19.5), and then aligned to the reference genome (human: GRCh38; mouse: GRCm39/mm39) using Bowtie2 (version 2.4.1), the reads were counted by FeatureCounts (version 2.0.6). Genes with differential expression across samples (differential expression genes, DEGs) were assessed using the “DESeq2” (version 1.42.0) package of R. An FDR (False Discovery Rate) of 0.05 and Log2 fold change cut‐off of 1 were imposed. GSEA was performed using the “enrichr” (version 3.2) and “GSEABase” (version 1.58.0) R packages. Gene sets were derived from MSigDB, including h.all.v2024.1.Hs.symbols.gmt, c2.cp.reactome.v2024.1.Hs.symbols.gmt, and c5.all.v2024.1.Hs.symbols.gmt. Scores for resident,^[^ ^17^ ^]^ nonresident,^[^ ^17^ ^]^ and exhaustion^[^ ^18^ ^]^ gene sets were calculated. Kaplan–Meier survival curves were generated using the “survival” R package (version 3.3.1), with statistical significance assessed by the log‐rank test. Plots were visualized using the “ggpirate” (version 0.1.2) and “ggplot2” (version 3.4.2) R packages. Publicly available transcriptome data were integrated into the analysis. The MMRF‐CoMMpass dataset (NCT01454297), containing bulk RNA‐seq data from BM samples of NDMM patients, was downloaded from UCSC Xena (https://xenabrowser.net/datapages/). After excluding samples with incomplete survival information, 844 MM patients were included in the final cohort. Additionally, transcriptome data from the GEO database (GSE136337; *n* = 436 NDMM samples)^[^ ^19^ ^]^ provided OS and progression‐free survival (PFS) information. Expression data from 34 MM cell lines were retrieved from the Human Protein Atlas database (https://www.proteinatlas.org/) for comparative analysis of immune checkpoint ligand expression.

### Single‐Cell RNA Sequencing and Data Analysis

BM and PB samples were collected from one RRMM patients on the 35th day post‐BCMA CAR‐T infusion. PBMCs and BMNCs were isolated using density gradient centrifugation with Ficoll (Sigma–Aldrich). CAR‐T cells were sorted based on CD3 and BCMA CAR expression via flow cytometry (BD Biosciences FACS Aria II) and subjected to single‐cell sequencing. Library preparation was conducted using the DNBSEQ technology platform and the DNBelab C4 Single‐Cell Library Prep Set.^[^ ^20^ ^]^ Paired‐end sequencing of single‐cell cDNA libraries was performed on the DIPSEQ T1 sequencing platform. The raw FASTQ files were subjected to quality control, read alignment, and feature quantification using the pipeline provided by DNBC4Tools (https://github.com/MGI‐tech‐bioinformatics/DNBelab_C_Series_HT_scRNA‐analysis‐software). Three single‐cell RNA‐seq datasets were integrated (GSE124310,^[^ ^5^ ^]^ GSE161801,^[^ ^21^ ^]^ and GSE223060^[^ ^22^ ^]^) comprising BMNCs from healthy individuals (NBM = 17) and 83 patients (SMM = 11;MGUS = 5; NDMM = 48; and RRMM = 19). The RRMM patients relapsed after standard regimens such as IMiDs, PIs, and anti‐CD38 antibodies, without exposure to BCMA‐targeted CAR‐T or BiTE therapies. The raw data for each dataset were processed to create individual Seurat (version 5.1.0) objects. Cell barcodes, gene names, and feature matrices were standardized across datasets, ensuring uniform annotation. Initial quality control metrics were calculated for each dataset. Individual datasets were merged using the merge function in Seurat after filtering. Principal component analysis (PCA) was performed on the top 2000 variable genes in merged datasets for dimensionality reduction. Harmony (version 1.2.1) was applied for batch correction and integration of multiple datasets. Using the Louvain algorithm based on shared nearest neighbors (SNN) for clustering. Uniform manifold approximation and projection (UMAP) and T‐distributed stochastic neighbor embedding (TSNE) were applied for visualization of cell clusters. Clusters were annotated based on known marker genes and enriched pathways. Identification of cluster‐specific marker genes using the FindMarkers function. Expression datasets for NK and Cytotoxicity,^[^ ^8b^ ^]^ Exhaustion,^[^ ^18^ ^]^ Adhesion,^[^ ^23^ ^]^ and Trm^[^ ^24^ ^]^ signature genes were queried (Table S9, Supporting Information), and module scores were calculated using AddModuleScore function. Gene or signature scores across cell populations were visualized using FeaturePlot function. At the single‐cell level, a CD8 T cell was defined as *CD69* high expression (*CD69* ^high^; TRM) if its expression > 0.5 (the median expression value of *CD69* gene); otherwise, the cell was defined as *CD69* low expression (*CD69* ^low^; Cir). Correlation analyses were performed using the Spearman algorithm. Similarly, cells with high *KLRB1* expression (according to the median of *KLRB1* expression) were classified as *KLRB1* ^high^ cells, and those with low *KLRB1* expression were categorized as *KLRB1* ^low^ cells. DEGs between MM, RRMM, and NBM were identified using the “DESeq2” (version 1.42.0) package. These DEGs were subjected to GSEA using the “clusterProfiler” package (version 4.4.4). Pseudotime trajectories were plotted using the “Monocle 3” (version 1.3.1) package. All visualizations and plots were generated using the “ggplot2” (version 3.4.2) and “pheatmap” (version 1.0.12) packages.

For validation, scRNA‐seq datasets were analyzed from various human hematological malignancies, including AML (GSE250077), DLBCL and FL (GSE182434),^[^ ^25^ ^]^ MCL (GSE184031), and HL (GSE245920).^[^ ^26^ ^]^ TSNE visualizations were generated using the RunTSNE function with perplexity values set to 30 or 15, depending on the dataset. For AML, cells with high *CD69* expression were classified as TRM, while those with low *CD69* expression were categorized as Tcir. In lymphoma datasets, TRM cells were identified as *CD69* ^high^ populations, whereas *CD69* ^low^ populations were designated as Tcir. For DLBCL and FL, *ITGAE* was selected as an additional marker to define TRM.

### Statistical Analyses

Statistical analyses between two groups were performed using a two‐tailed unpaired or paired Student's *t*‐test for parametric data or a two‐tailed Wilcoxon rank‐sum test for nonparametric data. All experiments were performed three times. Experimental data were presented as the mean ± standard deviation (SD), as detailed in the figure legends. Survival analyses for human and murine subjects were performed using the log‐rank test. All available specimens from enrolled participants were included in correlative analyses, and no data were excluded from the analysis. Statistical analyses and data visualization were conducted using GraphPad Prism (version 9.4.1) or RStudio (version 4.3.0). Statistical significance was defined as *p* < 0.05, with significance levels denoted as follows: *p* < 0.05 (*), *p* < 0.01 (**), *p* < 0.001 (***), *p* < 0.0001 (****), and “ns” indicating no significance.

## Conflict of Interest

The authors declare no conflict of interest.

## Author Contributions

L.W. and L.X. contributed equally to this work. L.W., L.X., and X.L. conceptualized the project and designed the experiments. S.T., Q.Z., and X.C. collected data. Y.Y., R.L., H.X., Q.C., J.Z., Y.L., E.W., and T.J. provided samples from patients. L.X., L.W., Y.Z., and Y.Y. performed the experiments. L.W. and L.X. analyzed the data. L.W. and L.X. wrote the paper. L.W., J.L., and X.L. supervised the project. All authors reviewed the results and approved the final version of the paper.

## Supporting information



Supporting Information

Supplemental Tables

## Data Availability

RNA‐seq and scRNA‐seq data that support the findings of this study have been deposited in the Gene Expression Omnibus under accession code GSE285162, GSE285163, GSE285164. The data that support the findings of this study are available in the Supporting Information of this article.
