# Supplementary material for: Targeting of the CD161 Inhibitory Receptor Enhances Bone‐Marrow‐Resident Memory CD8+ T‐Cell‐Mediated Immunity against Multiple Myeloma
Source: Adv Sci (Weinh). 2025 Aug 25;12(42):e10888. doi: 10.1002/advs.202510888 (PMC12622403; doi:10.1002/advs.202510888)
Supplement: Supplementary file 1 — Supporting Information [file ADVS-12-e10888-s001.docx]

**Supporting Information-supplement figures**

**Targeting of the CD161 Inhibitory Receptor Enhances Bone Marrow-Resident Memory CD8^+^ T Cell-mediated Immunity Against Multiple Myeloma**

*Liwen Wang ^1#^,* *Linzhi Xie ^1#^,* *Yi Zhou ^1^, Shiming Tan ^1^, Yan Yu ^1^, Qing Zhang ^1^, Xuefeng Chen ^1^, Yuhan Yan ^1^, Ruiheng Luo ^1^, Han Xiao ^1^, Qian Cheng ^1^, Jian Zhang ^1^, Ying Li ^1^, Erhua Wang ^1^, Tiebing Jiang ^1^, Jing Liu ^1^, Xin Li ^1*^*

**Figure S1**

**
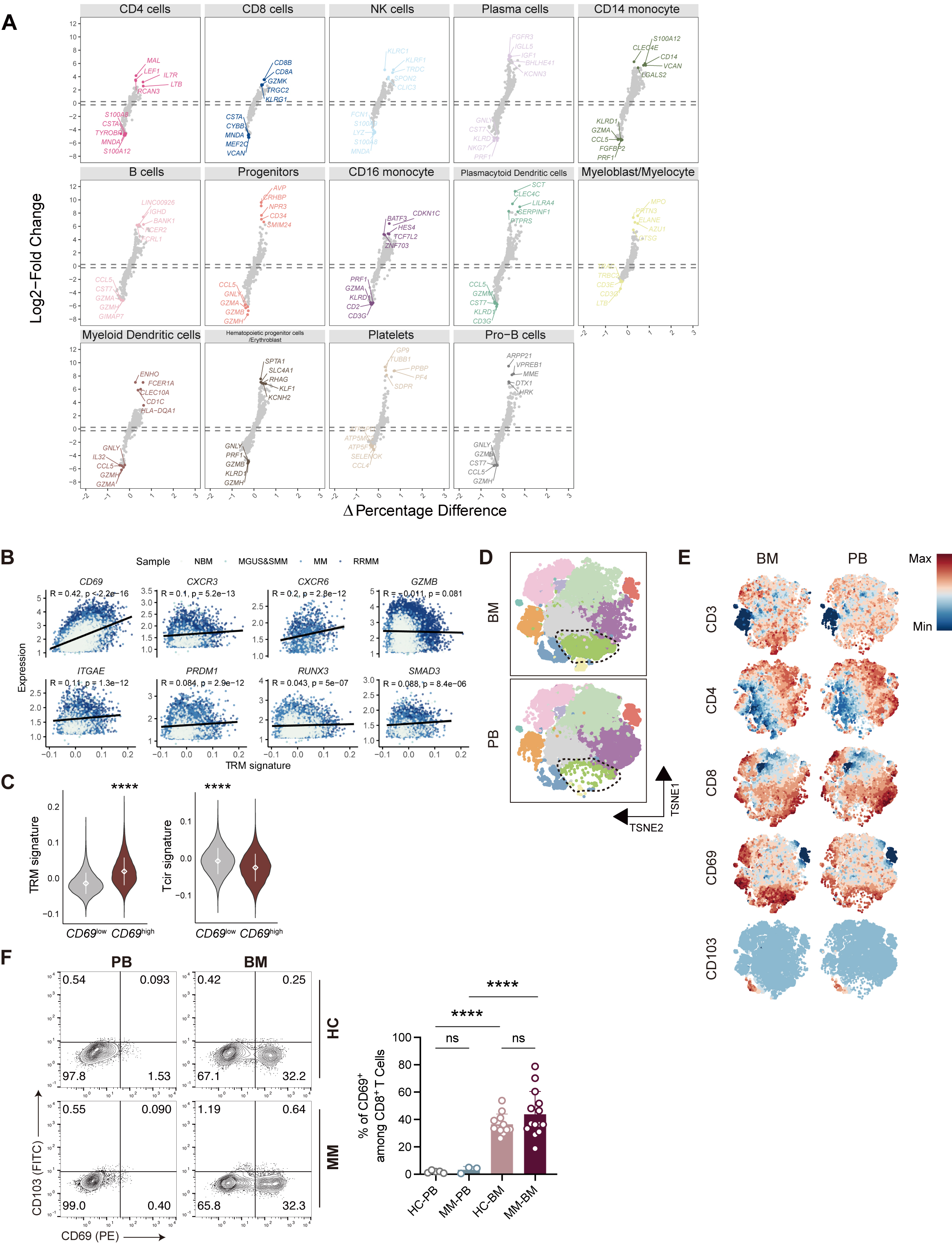
**

**Figure S2**

**
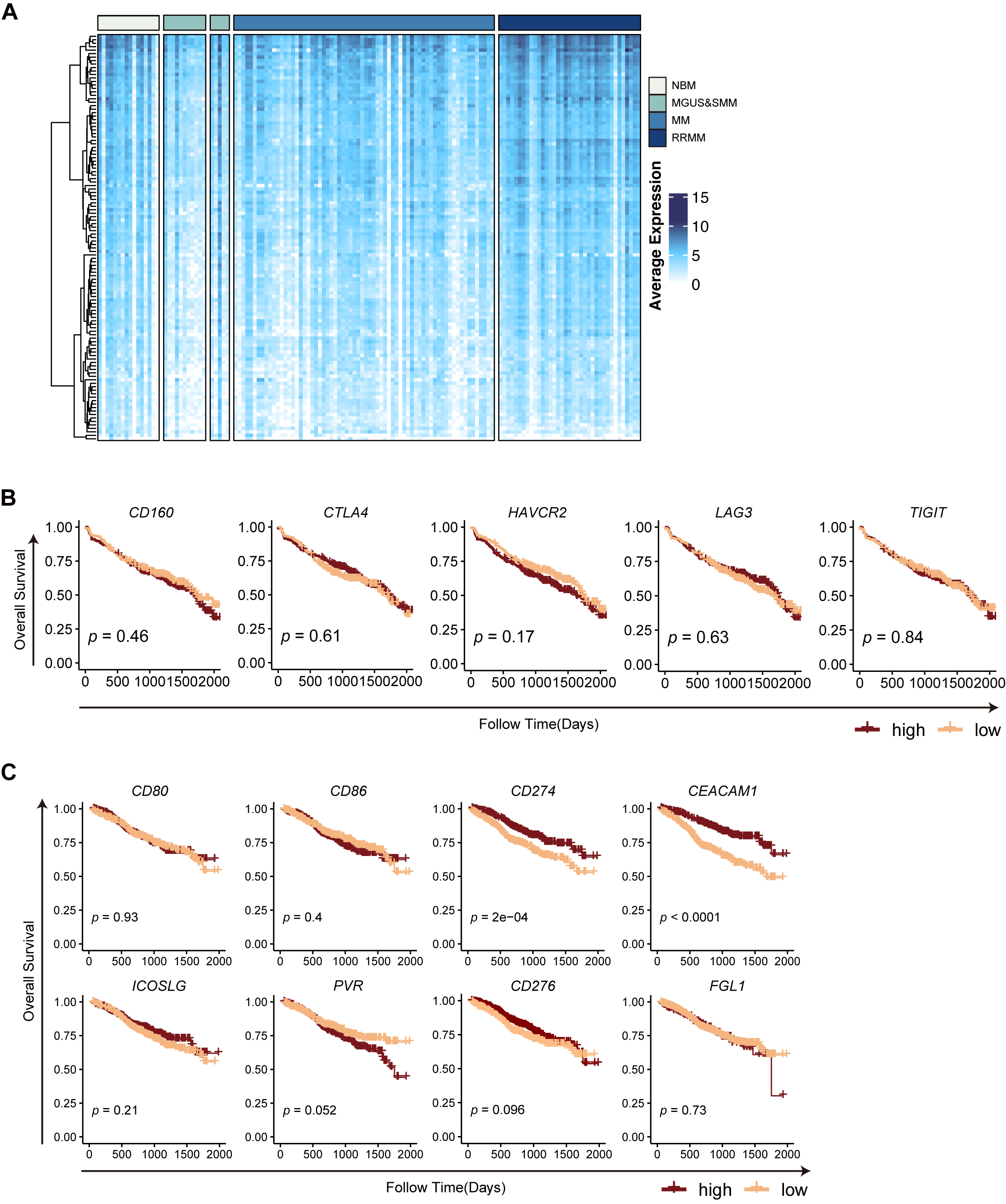
**

**Figure S3**

**
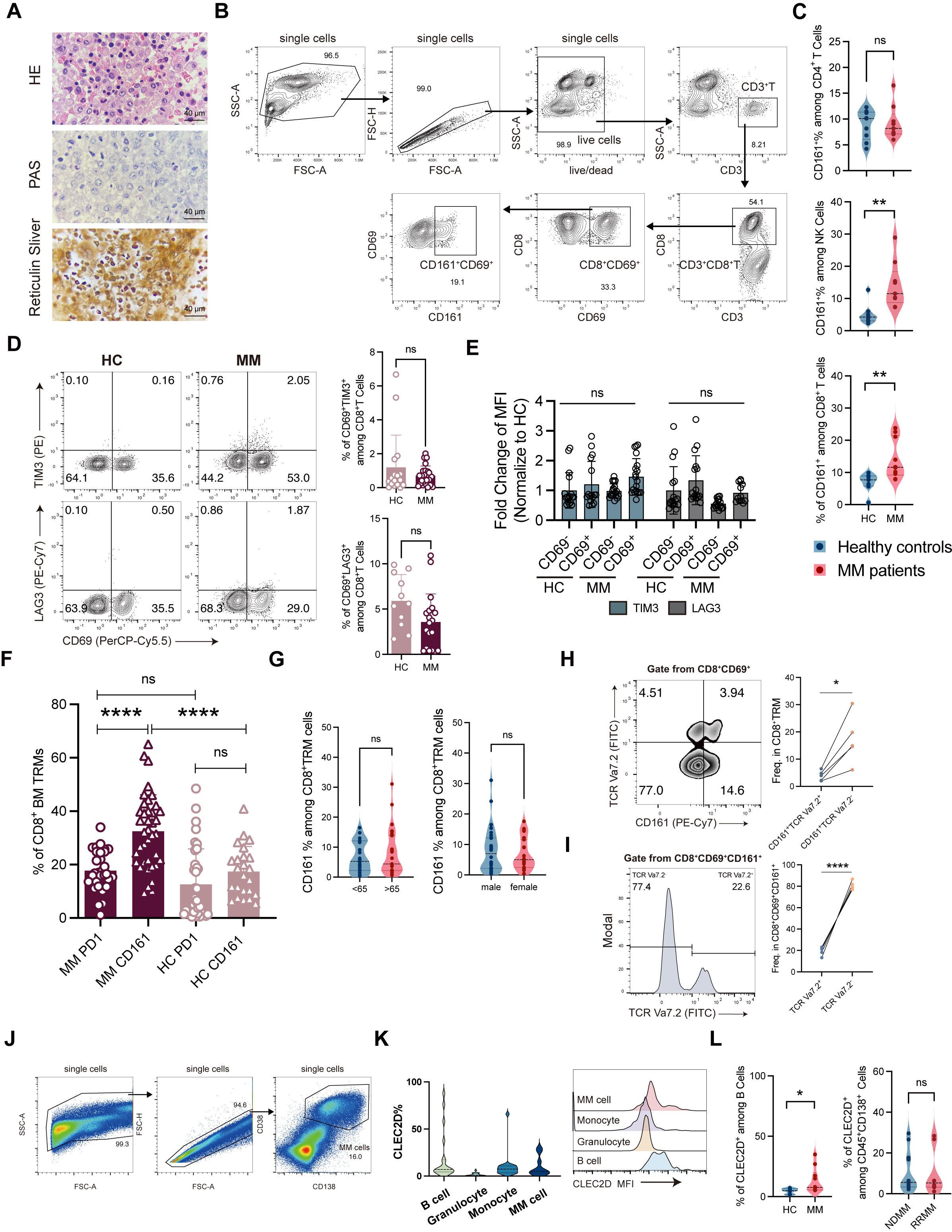
**

**Figure S4**

**
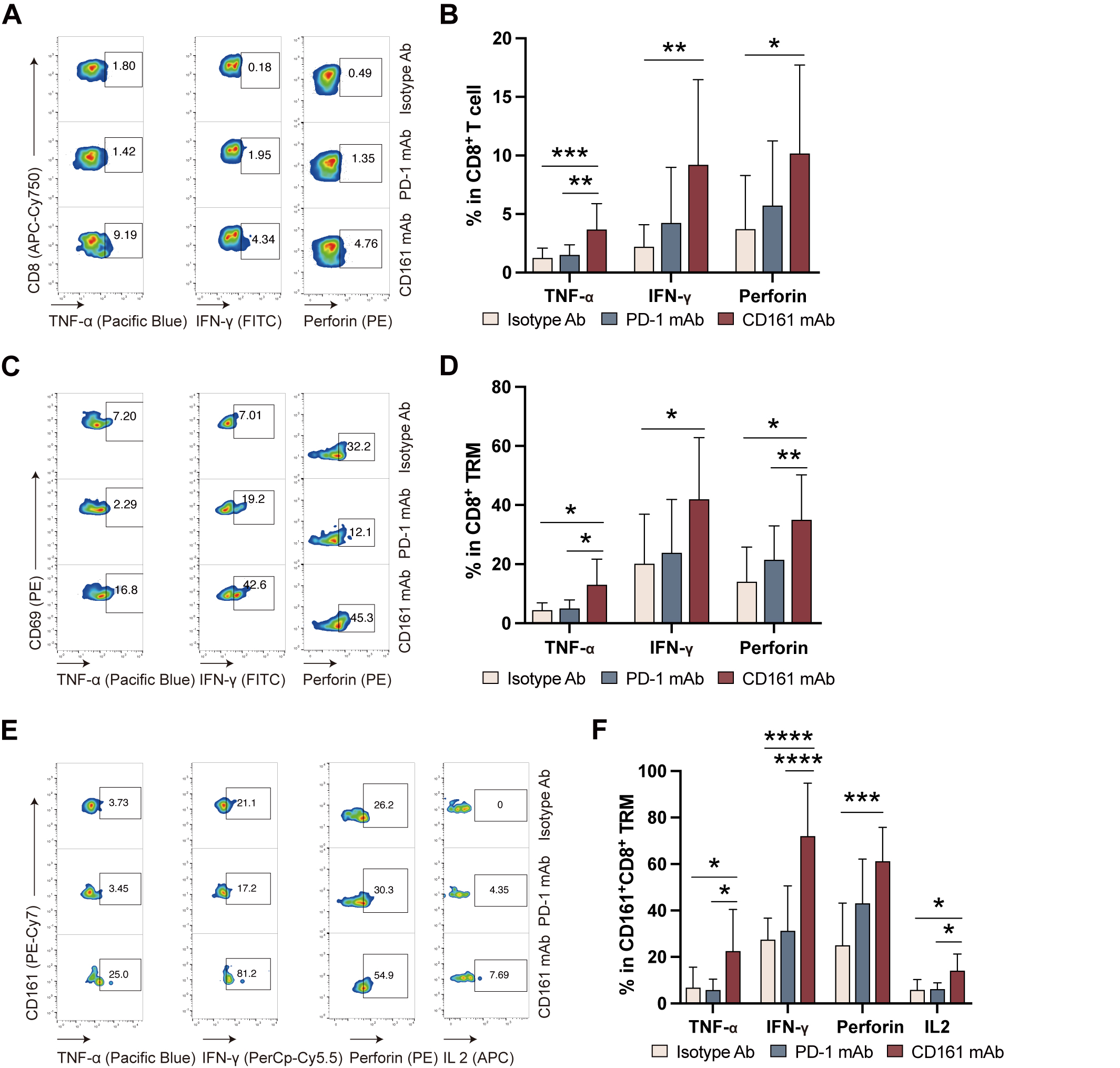
**

**Figure S5**

**
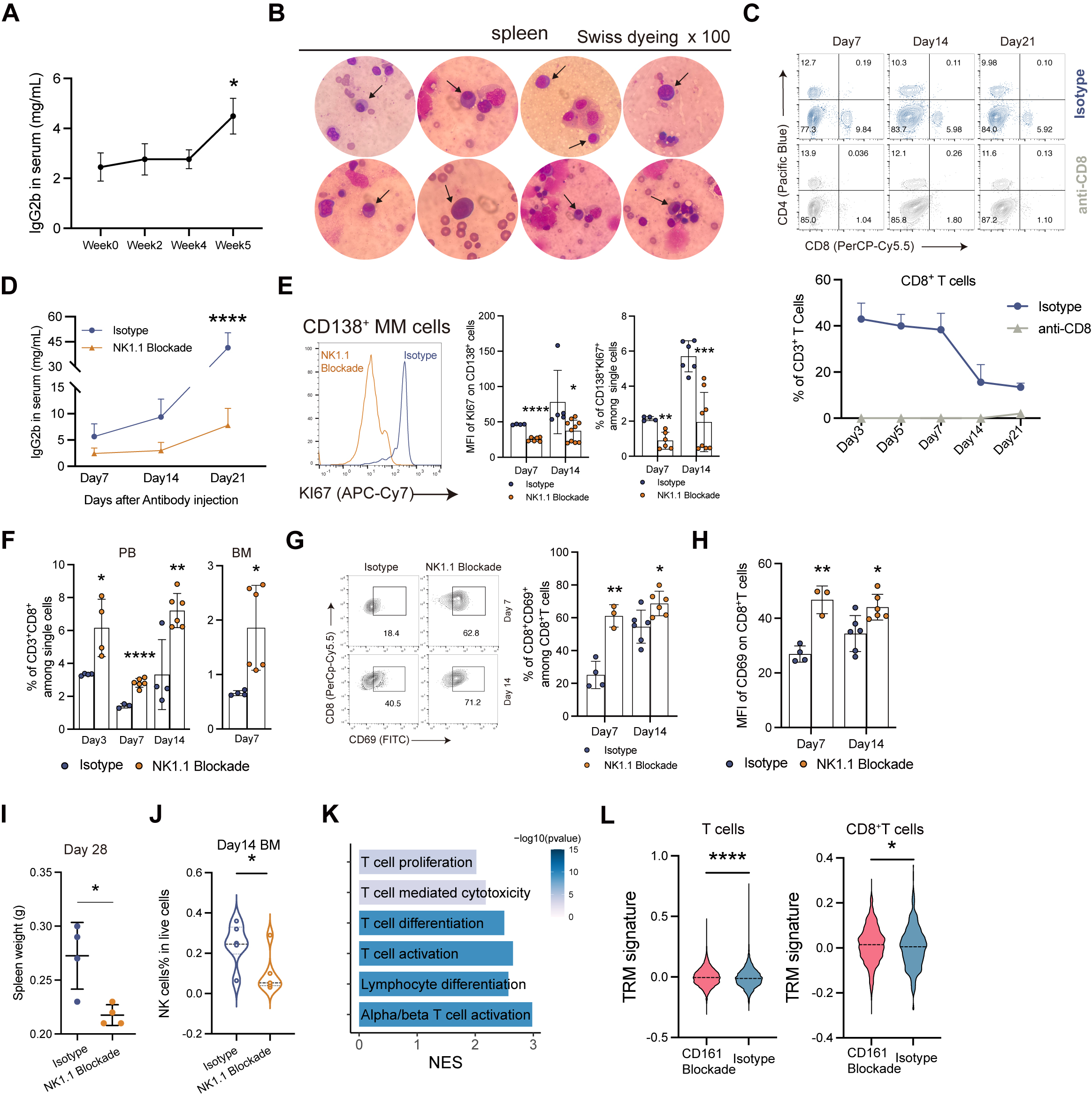
**

**Figure S6**

**
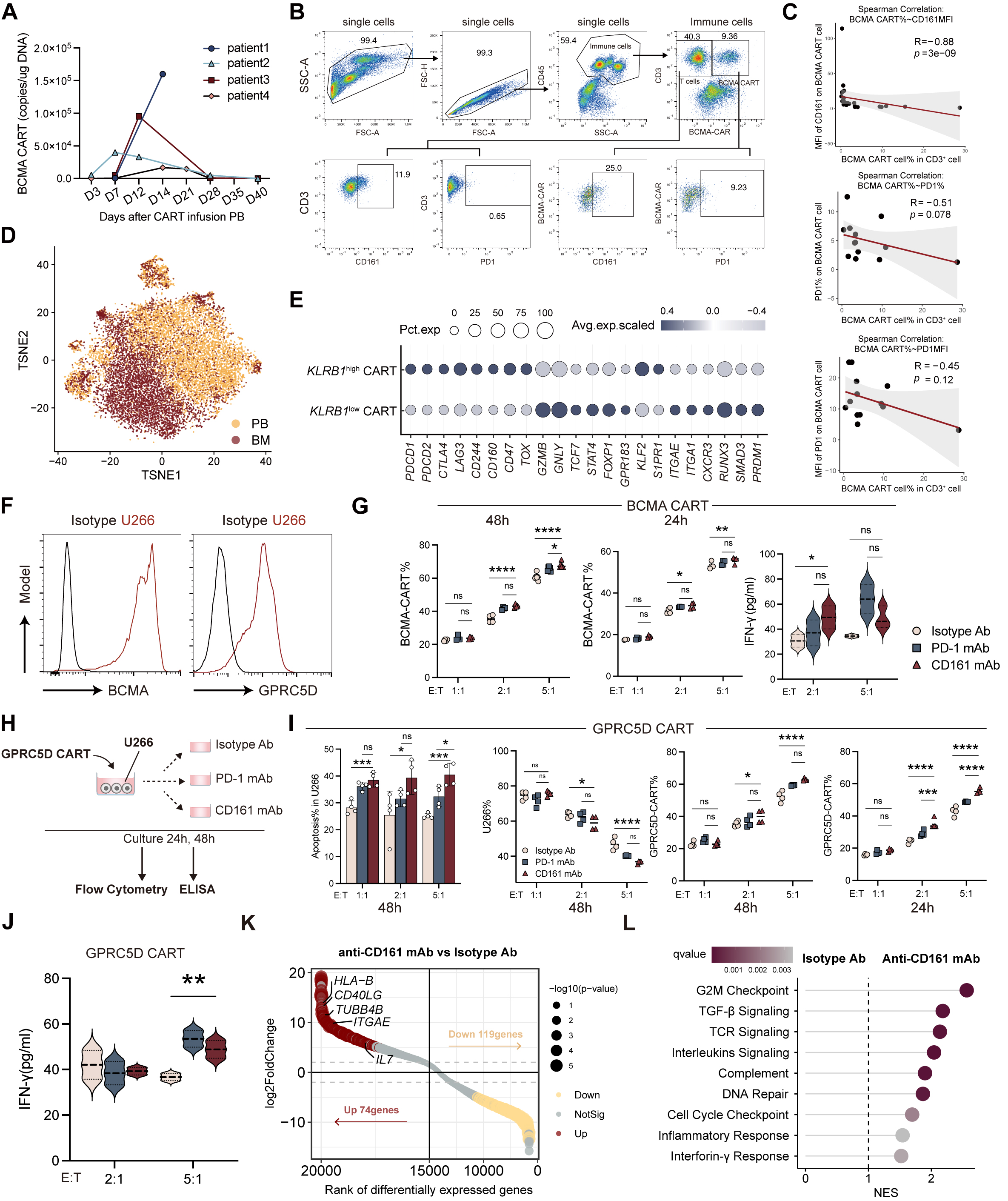
**

**Figure S7**

**
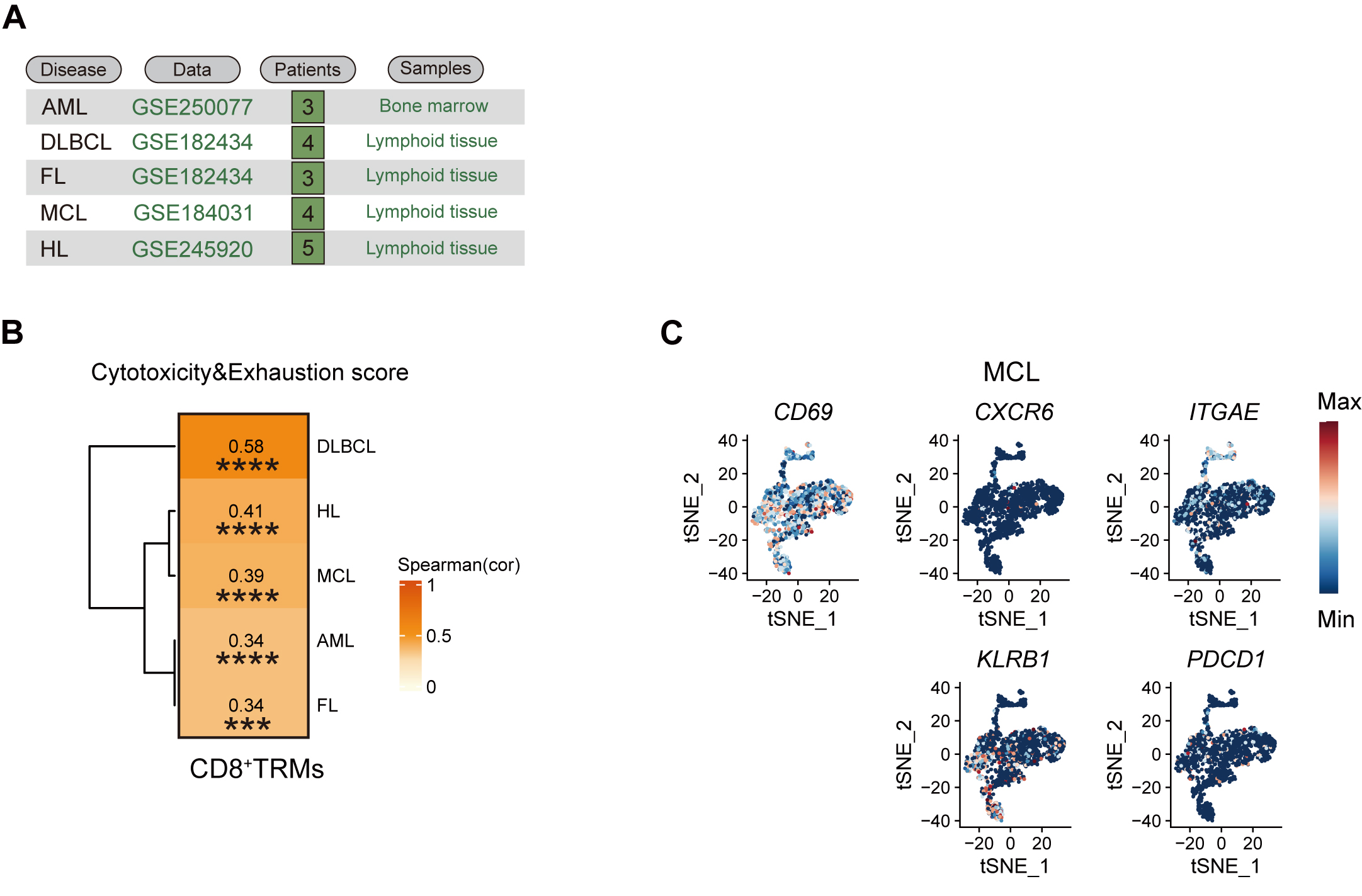
**

**Figure S1. Single cell sequencing to identify molecular markers of CD8^+^ TRM.**

**A)** Differential gene expression analysis using the log-fold change expression versus the difference in the percentage of cells comparing each cell type marker genes (△ Percentage Difference). Gene labeled have log-fold change>1, △ Percentage Difference>20% and P-value from Wilcoxon rank sum test<0.05. **B)** Spearman correlation analysis of TRM signature scores (x axis) versus mRNA levels of eight TRM core genes (y axis) for CD8^+^ T cells across NBM and each MM stage. **C)** Violin plots comparing TRM and Tcir signature scores between high and low *CD69-*expression populations in CD8^+^ T cells. Data were presented as mean ± SD. Significance was calculated with Student’s t-test. ****p < 0.0001. **D, E)** Representative TSNE plots showing T cells distributions in BM and PB from MM patients. **F)** Flow cytometry analysis of CD8⁺ T cells from BM(n=11) and PB(n=3) of HC and MM patients. Protein expression levels of CD69 (x axis) and CD103 (y axis) are shown (left). Bar plots compare CD69^+^ % among CD8^+^ T cells between HC and MM in PB and BM (right). Data were presented as mean ± SD. Significance was calculated with one way-ANOVA. ****p < 0.0001 and ns, no significance.

**Figure S2. Survival analysis of immune checkpoint ligand and receptor genes in the MM patient cohort.**

**A)** The heatmap shows the average expression level of NK signature genes in all samples at the NBM and MM stages. **B)** Kaplan–Meier curves showing the impact of high versus low expression of immune checkpoint receptor genes (*CD160*, *CTLA4*, *HAVCR2*, *LAG3*, *TIGIT*) on OS (GSE136337). *P* values were calculated using the Log-rank test. **C.** Kaplan–Meier curves showing the impact of high versus low expression of immune checkpoint ligand genes (*CD274*, *ICOSLG*, *CD80, CD86, CEACAM1, PVR*, *CD276*, *FGL1*) on OS (MMRF‐CoMMpass cohort). *P* values were calculated using the Log-rank test.

**Figure S3. CD161-CLEC2D axis represents a critical signaling pathway on CD8^+^ BM-TRM in MM.**

**A)** HE, Periodic Acid-Schiff (PAS), Reticulin sliver stained from MM BM biopsy sample. Scale bar: 40 μm. **B)** Gating strategies of CD161^+^CD69^+^CD8^+^T cells in MM BM by flow cytometry. **C)** Flow cytometry was used to analyze the expression level of CD161 on CD4^+^T cells, CD8^+^T cells and NK cells in BM of HC(n=9) and MM patients(n=9). Data were presented as mean ± SD. Significance was calculated with Student’s t-test. **p < 0.01 and ns, no significance. **D)** Flow cytometric analysis of CD69 (x axis), TIM3 (y axis) and LAG3 (y axis) on BM CD8^+^ T cells between HC(n=11) and MM patients(n=20) (left). Comparison of CD69^+^TIM3^+^ % and CD69^+^LAG3^+^ % among CD8^+^ T cells between HC and MM (right). Data were presented as mean ± SD. Significance was calculated with Student’s t-test. ns, no significance. **E)** Fold change of MFI for TIM3 and LAG3 on BM CD8^+^ T cells (classified as CD69^-^ or CD69^+^) from HC(n=11) and MM(n=20) was analyzed by flow cytometry (Normalized to HC CD69^-^CD8^+^ T cells). Data were presented as mean ± SD. Significance was calculated with one way-ANOVA. ns, no significance. **F)** Bar plots compare CD161^+^ % and PD1^+^ % among CD8^+^ TRM between HC(n=30) and MM(n=50) in BM. Data were presented as mean ± SD. Significance was calculated with one way-ANOVA. ****p < 0.0001 and ns, no significance. **G)** Frequencies of CD161% among CD8^+^ TRM were compared regarding age (left) and gender (right). Data were presented as mean ± SD. Significance was calculated with Student’s t-test. ns, no significance. **H)** Comparison of CD161^+^TCR Vα 7.2^+^% and CD161^+^TCR Vα 7.2^-^% among CD8^+^ TRM in MM BM(n=4). Data were presented as mean ± SD. Significance was calculated with Student’s t-test. *p < 0.05, ****p < 0.0001. **I)** Comparison of TCR Vα 7.2^+^% and TCR Vα 7.2^-^% among CD161^+^ CD8^+^ TRM in MM BM. **J)** Gating strategies of MM cells in MM BM by flow cytometry. **K)** Comparison of CLEC2D % (left) and MFI of CLEC2D (right) among several immune cells (B cell, Granulocyte, Monocyte) and MM cells. **L)** (left) CLEC2D expression frequency in B cells from HC and MM patients were evaluated by flow cytometry. (right) CLEC2D expression frequency in MM cells from NDMM and RRMM patients were evaluated by flow cytometry. Data were presented as mean ± SD. Significance was calculated with Student’s t-test.

**Figure S4. CD161 blockade enhances the function of CD8^+^ BM-TRM in MM.**

**A-F)** Flow cytometry analysis of the expression of TNF-α, IFN-γ, Perforin and IL-2 after treated with isotype(n=12), PD1 mAbs(n=12), and CD161 mAbs(n=12) in CD8^+^ T cells (**A, B**), CD8^+^ TRM (**C, D**), CD161^+^CD8^+^ TRM (**E, F**). Data were presented as mean ± SD. Significance was calculated with one way-ANOVA. *p<0.05, **p<0.01, ***p<0.001 and ****p<0.0001.

**Figure S5. NK1.1 blockade improves survival in MM mice models.**

**A)** Line plots depict serum IgG_2b_ levels from the PB of mice during the modeling period (week 0 to week 5, n=3 mice). Asterisks * indicates statistically significant differences between week 4 and week 5. **B)** Swiss dyeing stained from mice spleens. The image is magnified 100 times by light microscope, and the black arrow points to tumor cells. **C)** Flow cytometry dot plots show expression of CD4 and CD8 in CD45^+^cells in PB of isotype and CD8 depletion group mice at day 7, 14, 21 (up). Line graphs show the proportion of CD8^+^T cells among CD3^+^T cells in PB of mice on days 3,5,7,14, and 21 after antibody treatment (bottom). **D)** Line plots show serum IgG_2b_ levels from PB in two groups (NK1.1 blockade and isotype) after antibody administration (n=3 mice). Data were presented as mean ± SD. Significance was calculated with Student’s t-test. ****p < 0.0001. **E)** Comparison of CD138^+^KI67^+^ % among single cells from BM between isotype and NK1.1 blockade on days 7, 14. Data were presented as mean ± SD. Significance was calculated with Student’s t-test. *p < 0.05, **p < 0.01, ***p < 0.001, ****p < 0.0001. **F)** Flow cytometry analysis of CD3^+^CD8^+^ % among single cells in PB, BM on days 3, 7,14 post antibody injection. Data were presented as mean ± SD. Significance was calculated with Student’s t-test. *p < 0.05, **p < 0.01, ****p < 0.0001. **G)** Flow cytometry analysis of CD8^+^CD69^+^% among CD8^+^T cells in BM on days 7,14 post antibody injection. Data were presented as mean ± SD. Significance was calculated with Student’s t-test. *p < 0.05, **p < 0.01. **H)** Flow cytometry analysis of MFI of CD69 on CD8^+^T cells in BM on days 7,14 post antibody injection. Data were presented as mean ± SD. Significance was calculated with Student’s t-test. *p < 0.05, **p < 0.01. **I)** The weight of spleens from mice (n=4) treated with isotype and NK1.1 blockade on day 21. Data were presented as mean ± SD. Significance was calculated with Student’s t-test. *p < 0.05. **J)** Flow cytometry analysis of NK cells % among live cells in BM on days 14 post antibody injection. Data were presented as mean ± SD. Significance was calculated with Student’s t-test. *p < 0.05. **K)** Gene set enrichment analysis of DEGs (NK1.1 blockade vs isotype) was performed using MSigDB gene sets (c5). **L)** Violin plot compares TRM signature scores between CD161 blockade and isotype in total T cells and CD8^+^ T cells. Data were presented as mean ± SD. Significance was calculated with Student’s t-test. *p < 0.05, ****p < 0.0001.

**Figure S6. Dynamic regulation of CD161 during late-stage CAR-T cell expansion drives exhaustion and impacts antitumor efficacy.**

**A)** Dynamic changes of CART copies in MM patients (n=4) receiving BCMA CART at the indicated time points by qPCR. **B)** Gating strategies of CD161% and PD1% among CART cells in MM PB by flow cytometry. **C)** Spearman correlation analysis of MFI of CD161 in BCMA CART cells (top, y axis, n=24), PD1% in BCMA CART cells (center, y axis, n=13), and MFI of PD1 in BCMA CART cells (bottom, y axis, n=13) with BCMA CART% (x axis) in MM patients. Significance was calculated with Spearman test. **D)** T-SNE plot illustrating the distribution of BCMA CART cells in BM and PB. **E)** Bubble plot displaying expression levels of exhaustion, cytotoxicity, anti-resident, and pro-resident genes in two clusters (*KLRB1*^high^CART and *KLRB1*^low^CART). **F)** Protein expression levels of BCMA and GPRC5D in U266 cell line, with isotype control antibody staining shown in black. Isotype antibody staining of U266 is used as a negative control. **G)** Comparison of BCMA CART cells% across three treatment groups (isotype, CD161 mAb, PD-1 mAb) after 24- or 48-hours culture at different E: T (1:1, 2:1, 5:1) ratios. IFN-γ in the supernatant was quantified by ELISA from BCMA CART cells (right) (n=3). Data were presented as mean ± SD. Significance was calculated with one way-ANOVA. *p < 0.05, **p < 0.01, ****p < 0.0001 and ns, no significance. **H)** Experimental design. **I)** Comparison of U266 cells%, apoptosis% in U266, and CART cells% across three treatment groups (isotype, CD161 mAb, PD-1 mAb) for GPRC5D CART cells after 24- or 48-hours culture at different E: T (1:1, 2:1, 5:1) ratios. Data were presented as mean ± SD. Significance was calculated with one way-ANOVA. *p < 0.05, ***p < 0.001, ****p < 0.0001 and ns, no significance. **J)** IFN-γ in the supernatant was quantified by ELISA from GPRC5D CART cells (right) (n=3). Data were presented as mean ± SD. Significance was calculated with one way-ANOVA. **p < 0.01. **K)** Volcano plot of significant DEGs in BCMA CART cells comparing anti-CD161 to isotype (*p* value<0.01, |log2FC|>1). **L)** Gene set enrichment analysis of BCMA CART DEGs (anti-CD161 vs isotype) using gene sets for MSigDB (Hallmark and Reactcome).

**Figure S7. Identification of a *KLRB1*-driven exhaustion program in CD8^+^ TRM across** **hematological malignancies.**

**A)** Schematic depicting the hematological malignancies datasets and detailed information. **B)** Heatmap shows spearman correlation between cytotoxicity scores and exhaustion scores in CD8^+^ TRM across hematological malignancies tissues. Asterisk stands for *p* value. ***p < 0.001, ****p < 0.0001. Significance was calculated with Spearman test. **C)** TSNE visualization illustrating the expression of representative genes in CD8^+^ T cells isolated from MCL lymphoid tissue.
